# ABC transporters linked to multiple herbicide resistance in blackgrass (*Alopecurus myosuroides*)

**DOI:** 10.3389/fpls.2023.1082761

**Published:** 2023-03-17

**Authors:** Alina Goldberg-Cavalleri, Nawaporn Onkokesung, Sara Franco-Ortega, Robert Edwards

**Affiliations:** Agriculture, School of Natural and Environmental Science, Newcastle University, Newcastle Upon Tyne, United Kingdom

**Keywords:** compartmentation, detoxification, non-target site herbicide resistance, wild grasses, vacuole, xenobiotics

## Abstract

Enhanced detoxification is a prominent mechanism protecting plants from toxic xenobiotics and endows resistance to diverse herbicide chemistries in grass weeds such as blackgrass *(Alopecurus myosuroides*). The roles of enzyme families which impart enhanced metabolic resistance (EMR) to herbicides through hydroxylation (phase 1 metabolism) and/or conjugation with glutathione or sugars (phase 2) have been well established. However, the functional importance of herbicide metabolite compartmentalisation into the vacuole as promoted by active transport (phase 3), has received little attention as an EMR mechanism. ATP-binding cassette (ABC) transporters are known to be important in drug detoxification in fungi and mammals. In this study, we identified three distinct C-class ABCCs transporters namely *Am*ABCC1, *Am*ABCC2 and *Am*ABCC3 in populations of blackgrass exhibiting EMR and resistance to multiple herbicides. Uptake studies with monochlorobimane in root cells, showed that the EMR blackgrass had an enhanced capacity to compartmentalize fluorescent glutathione-bimane conjugated metabolites in an energy-dependent manner. Subcellular localisation analysis using transient expression of GFP-tagged *Am*ABCC2 assays in *Nicotiana* demonstrated that the transporter was a membrane bound protein associated with the tonoplast. At the transcript level, as compared with herbicide sensitive plants, *AmABCC1* and *AmABCC2* were positively correlated with EMR in herbicide resistant blackgrass being co-expressed with *AmGSTU2a*, a glutathione transferase (GST) involved in herbicide detoxification linked to resistance. As the glutathione conjugates generated by GSTs are classic ligands for ABC proteins, this co-expression suggested *AmGSTU2a* and the two ABCC transporters delivered the coupled rapid phase 2/3 detoxification observed in EMR. A role for the transporters in resistance was further confirmed in transgenic yeast by demonstrating that the expression of either *Am*ABCC1 or *Am*ABCC2, promoted enhanced tolerance to the sulfonylurea herbicide, mesosulfuron-methyl. Our results link the expression of ABCC transporters to enhanced metabolic resistance in blackgrass through their ability to transport herbicides, and their metabolites, into the vacuole.

## 1 Introduction

In northern Europe, blackgrass (*Alopecurus myosurides* [Huds.]) is a problematic weed of wheat and other arable crops, with many populations having evolved resistance to multiple herbicides (Moss et al., 2007). Without effective control, herbicide-resistant blackgrass incurs very significant crop losses, causing an estimated loss of 0.8 million tons of the annual winter wheat harvest, with a value of £0.5 billion, in the UK alone (Varah et al., 2020). With the incidence of herbicide resistance in wild grasses becoming more widespread across the globe, an increasing amount of effort is being expended on understanding the evolution of this damaging trait and the underpinning resistance mechanisms.

**Figure 1:**
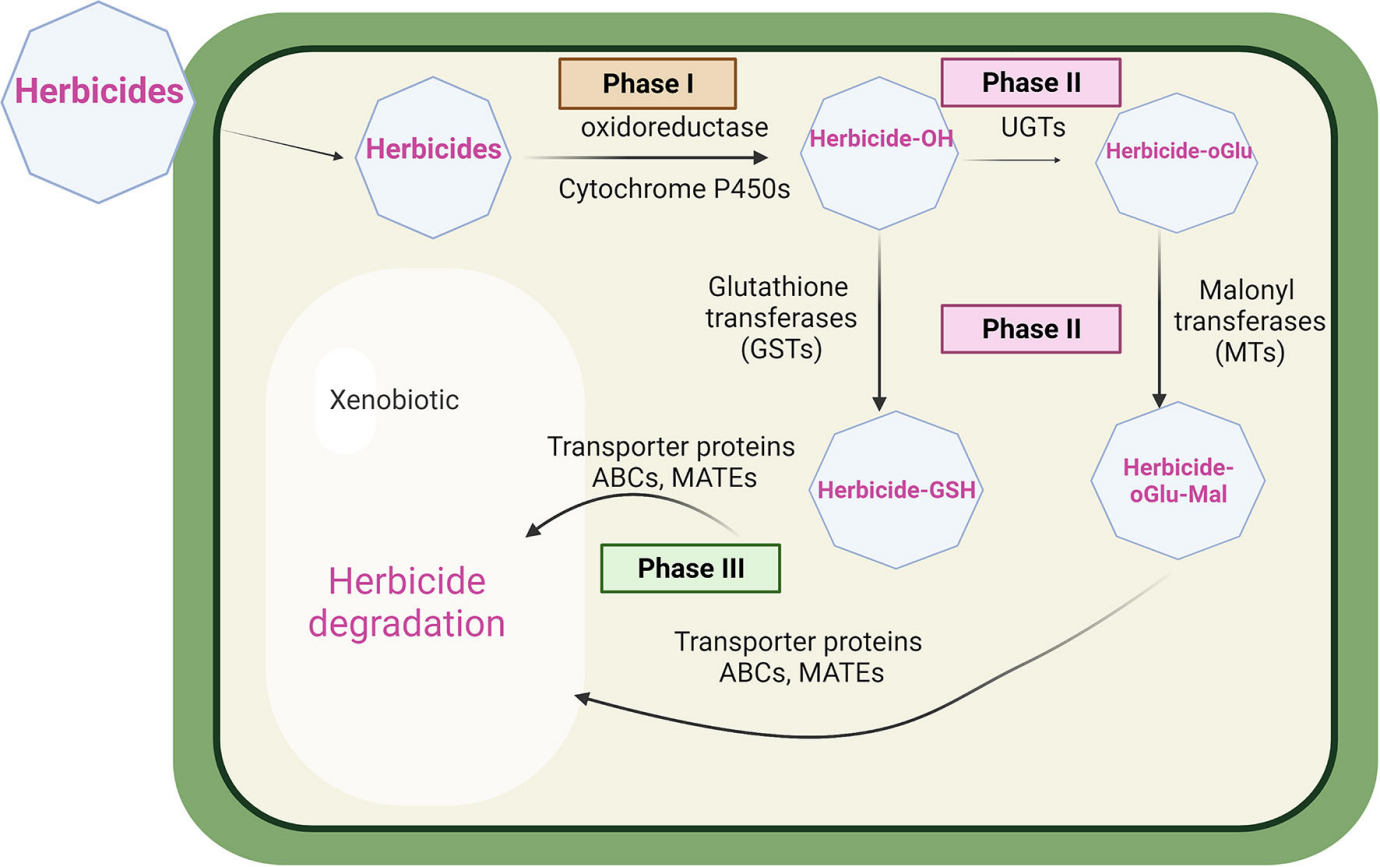
Herbicide detoxification in plants. In phase 1, herbicides are typically metabolized by cytochrome P450 monooxygenase enzymes prior to phase 2 bioconjugation with glutathione or sugars catalysed by glutathione transferase (GSTs), or UDP-glycosyl dependent transferases (UGTs) malonyl transferases (MTs). In phase 3, conjugated metabolites are then transported into the vacuole by ABC, or MATE transporters, pending further degradation.

In terms of global mechanisms, two types of herbicide resistance, notably target site resistance (TSR) and non-target site resistance (NTSR) have been described in both dicot and monocot weeds (Délye, 2013; Gaines et al., 2020). TSR endows resistance to herbicides at their site of action, through point mutations giving rise to protein targets that have a reduced sensitivity to chemical inhibition (Powles and Yu, 2010; Gaines et al., 2020). While TSR is extremely efficient in conferring resistance to herbicides with that mode of action, it does not extend to other classes acting on other target sites. In contrast, non-target site resistance (NTSR) is a polygenic adaptive trait derived from diverse molecular mechanisms conferring resistance to multiple herbicides, that does not involve in modifications to the target site. NTSR is quantitative, extending from modest to high levels of conferred resistance to herbicides which can vary in their chemistry and mode of action (Petit et al., 2010; Délye et al., 2013). While the molecular mechanisms underpinning NTSR are complex and not fully understood, enhanced metabolism-based resistance (EMR) is a major contributing factor (Délye et al., 2013). For EMR in weeds, the rates of herbicide detoxification are accelerated through the coordinated upregulation of enzymes involved in primary metabolic activation, notably cytochromes P450 monooxygenases (CYPs) and secondary bioconjugation catalysed by glutathione transferases (GSTs) or UDP-sugar-dependent glycosyltransferases (UGTs) (Edwards et al., 2005; Yu and Powles, 2014). As such, EMR in plants closely resembles a similar coordinated upregulation of detoxification enzymes underpinning multiple drug resistance (MDR) in animals and fungi (Housman et al., 2014; Cheng et al., 2022). In MDR, in addition to the upregulation of detoxification enzymes, energy-dependent membrane transporters are also enhanced, allowing for the accelerated cellular export of conjugated detoxification products, typically leading to extracellular extrusion (Choi and Yu, 2014). This active transport is typically achieved through the action of ATP-binding cassette (ABC) transporters. Such efflux is critical to maintaining efficient drug detoxification, as otherwise metabolic intermediates can build up in the cell and lead to feedback inhibition (Gottesman et al., 2002). A schematic showing the herbicide detoxification system present in plant cells is shown in **Figure 1**.

ABC transporters are one of the largest protein families conserved across the kingdoms and contain transmembrane domains (TMDs) responsible for the transport of a wide range of substrates across membranes through a mechanism driven by ATP hydrolysis (Higgins, 1992; Henikoff et al., 1997). ABC proteins comprise two TMDs and two nucleotide-binding domains (NBD)s. The NBDs are responsible for ATP binding and hydrolysis, while each TMD contains six transmembrane helices forming a translocation pathway across the membrane bilayer which incorporates a ligand-binding site (Higgins, 1992). While TMDs generally share a similar topology within a transporter class, they display no significant sequence conservation, which have allowed ABC proteins to evolve the ability to transport a broad range of substrates (Wilkens, 2015). ABC transporter can act as either importers or exporters, with ATP binding and hydrolysis at the NBDs altering the conformations of the TMDs which control ligand access and determine the direction of transport across the lipid bilayer (Higgins, 1992; Biemans-Oldehinkel et al., 2006; Beis, 2015). In eukaryotic organisms, ABC transporters can be classified into nine subfamilies (ABCA-ABCI), based on domain organizations and primary sequence homology (Dean and Annilo, 2005; Verrier et al., 2008).

In plants, many coding sequences for ABC proteins have been identified evidencing a multiplicity of these transporters, though only a small number have been functionally characterised. For instance, Arabidopsis (*Arabidopsis thaliana)* and rice (*Oryza sativa*) each contain approximately 130 coding sequences for ABC proteins (Sánchez-Fernández et al., 2001; Verrier et al., 2008; Hwang et al., 2016). The large number of ABC transporters has been linked to the diverse range of endogenous metabolites requiring transmembrane transport as plants respond to an ever-changing environment (Lefèvre et al., 2015; Hwang et al., 2016). Plant ABC transporters have been identified in the plasma membrane, and the membranes encapsulating the tonoplast, chloroplasts, mitochondria, and peroxisomes (Jaquinod et al., 2007; Verrier et al., 2008; Francisco et al., 2013). It has been proposed that the localisation of ABC transporters in different membranes associated with their specific functions such as the transportation of phytohormones or bioactive flavonoids (Goodman et al., 2004; Kuromori et al., 2010; Francisco et al., 2013), cell wall components contributing to cuticle and pollen coat deposition (Choi et al., 2014), and defence compounds active against pathogens (Krattinger et al., 2009; Bienert et al., 2012).

In addition to their role in transporting endogenous metabolites, a small number of studies have also shown that ABC proteins are active in the compartmentation and detoxification of xenobiotics. For instance, glutathione-conjugated metabolites of *N-*ethylmaleimide (NEM-GS), the chloroacetanilide herbicide metolachlor (met-GS), 2,4-dinitrophenyl (DNP-GS), and the sulfonylurea herbicide chlorimuron-ethyl (CE-GS) were shown to be transported into the vacuoles of mung bean (*Vigna radiata)*, barley (*Hordeum vulgare)*, and beet (*Beta vulgaris)* in an Mg^2+^-ATP dependant manner (Martinoia et al., 1993; Li et al., 1995; Bartholomew et al., 2002). Expression of the Arabidopsis ABC transporters (*AtMPR1* and *AtMPR2*) in yeast was associated with the transport of met-GS and DNP-GS into the vacuoles of transgenic cells (Lu et al., 1997; Lu et al., 1998). Such studies have tended to focus on the roles for plant ABC proteins in the phytoremediation of xenobiotics, or herbicide metabolism in crops. In contrast, the functional roles of ABC transporters in EMR mechanisms in weed species have received less attention. In a recent study with the C-class *Ec*ABCC8 in jungle rice (*Echinochloa colona)*, this ABC transporter was shown to actively efflux glyphosate out of plant cells, resulting in resistance to the herbicide as demonstrated in both jungle rice and in transgenic rice overexpressing the protein (Pan et al., 2021). However, the role of ABC proteins in EMR to additional herbicides were resistance is linked to further detoxification has not been reported.

In this study, we have investigated the involvement of ABC transporters in NTSR and EMR in the problem weed blackgrass. The approach adopted has been to identify potential ABC proteins of interest using global transcript expression analysis of NTSR and herbicide sensitive (HS) blackgrass populations (Tétard-Jones et al., 2018). The respective full length coding sequences of proteins have then been assembled and the expression of the respective genes and proteins studied *in planta*, and the functioning of the transporters then determined in transgenic yeast cells. Of particular interest has been to study how these transporters function with xenobiotic metabolising enzymes as an integrated detoxification system that underpins NTSR and EMR (**Figure 1**).

## 2 Materials and methods

### 2.1 Identification of ABC transporters in blackgrass

To identify potential transporter genes encoding ABC and multidrug and toxic compound extrusion (MATE) proteins, contig sequences from the NTSR blackgrass transcriptome were assembled (Tétard-Jones et al., 2018) and compared to transporter sequences deposited in the National Centre for Biotechnology Information (NCBI) database (https://www.ncbi.nlm.nih.gov/) using the online protein Blast (BlastP) analysis tool (https://blast.ncbi.nlm.nih.gov/Blast.cgi). The sequences of ABCC transporters were confirmed by amplifying the respective open reading frames (ORFs) by polymerase chain reaction (PCR) using cDNA prepared from NTSR blackgrass using specific primers from untranslated regions (UTR). The amplified fragments were cloned in pGEM-T Easy Vector (Promega) and the ORFs were sequenced using specific primers (**Supplementary Table S1**). A topology model of the *Am*ABC1-3 membrane proteins was based on the TOPCONS consensus sequence (Tsirigos et al., 2015) and models were visualised with Protter (Omasits et al., 2014), with NBD alignments generated with SEAVIEW software version 4.6.4 (Gouy et al., 2010) using Muscle (Edgar, 2004).

For phylogenetic analysis, alignments of novel ABC sequences from blackgrass and homologues from other species were generated within SEAVIEW software version 4.6.4 (Gouy et al., 2010) using Muscle (Edgar, 2004) and subsequently trimmed (trimAI software v.1.3) using the settings ‘gappyout’ (Capella-Gutíerrez et al., 2009), as accessed through the webserver Phylemon 2 (Sánchez et al., 2011). Trimmed alignments were used for infer maximum likelihood phylogenies with IQ-TREE software (Nguyen et al., 2015). Automatic model selection mode was used (Bayesian information Criterion selecting the model LG+G4+I+F) and branch support values calculated by Ultrafast bootstrap approximation. The phylogenetic tree was edited with iTOL tool (https://itol.embl.de/) as described in Letunic and Bork, 2021.

### 2.2 Plant material and growth conditions

The herbicide resistance profiles of blackgrass populations used in this study were as previously described (Moss, 1990; Moss et al., 2007; Marshall et al., 2013; Sabbadin et al., 2017). The sources and resistance profile of each population are summarized in **Table S3**. Blackgrass seeds were germinated and grown as described previously (Franco-Ortega et al., 2021).

*Nicotiana benthamiana* seeds were surfaced sterile with 5% sodium hypochlorite containing 0.1% (v/v) Tween 20 for 5 min. Seeds were washed (5x) with sterile deionised water and placed on MS media containing 3% (w/v) sucrose. Plates were kept in the dark at 4°C for 2 d before being transferred to a growth cabinet at 21 ± 1°C; 120 μmol m^–2^ s^–1^ light intensity, and a 16 h:8 h (light: dark) photoperiod for 14 d. Seedlings were transferred to plastic pots (10 cm diameter) containing John Innes Number 2 compost and maintained under the same environmental conditions.

### 2.3 *In vivo* study with monochlorobimane in blackgrass roots

A solution of aqueous monochlorobimane (MCB; 100 µM) and propidium iodide (PI; 50 µM) was pipetted on a microscope glass slide, with 5µM sodium azide (NaN_3_) added to provide a negative control. Blackgrass roots from 5 d old seedlings were placed in the solutions under coverslips. Images were acquired by SP8 invert confocal microscope (Leica microsystem) using 20x lens at 5-, 10-, 15- and 20-minutes intervals with excitation at 442 nm for the glutathione-bimane (GSB) conjugate and 480 nm for the PI. Fluorescence intensity of the GSB in individual cells was determined using LASX software (Leica microsystem) with 20 cells (n = 20) individually quantified and experiments repeated twice with independent samples. Data were analysed by Student’s *t-*test, using SPSS 27 (IBM, Chicago, IL, USA).

### 2.4 Herbicide treatments

Herbicides were obtained from Sigma Aldrich (Gillingham, UK) and stock solutions prepared in dimethyl sulfoxide (DMSO). Four- week-old (3-5 leaf) HS or NTSR blackgrass plants were treated with 40 μM mesosulfuron-methyl, or 85 μM clodinafop-propargyl, with 0.1% (v/v) DMSO used as a solvent control. After a 24 h treatment, meristem and leaf tissue were separately harvested from individual plants (n = 7), then flash-frozen in liquid nitrogen and stored at -80°C.

### 2.5 Quantitative real-time PCR

Total RNA was isolated from ~100 mg of finely ground plant tissue using a NucleoSpin RNA plant kit following the manufacturer’s protocol (Macherey-Nagel, Germany). cDNA was synthesized from 1 μg of RNA using an iScript cDNA synthesis kit (Bio-Rad, United Kingdom) in a 20 μl reaction volume. RT-qPCR was performed in Light Cycler 96 system (Roche, United Kingdom) in a total volume of 15 μl containing 3.75 μl of cDNA,7.5 μl of LightCycler FastStart DNA Master SYBR Green I (Roche, United Kingdom), and 0.6 μl of 10 μM forward and reverse gene-specific primers. The primer sequences used in this study are listed in **Supplementary Table S1**. The reactions were run in a three-step program including melting curve; pre-incubation at 95°C for 3 minutes; amplification over 45 cycles (95°C for 10 s, 60°C for 10 s, and 72°C for 20 s; and melting analysis from 65°C to 95°C). For normalization, specific primers directed toward glyceraldehyde-3-phosphate dehydrogenase from blackgrass (*Am*GADPH; accession number: JN599100) were used, with relative gene expression (2^–ΔΔCt^) then calculated (Livak and Schmittgen, 2001; Pfaffl, 2001).

### 2.6 Antisera generation and protein immunoblot analysis

Synthetic peptides were prepared from gene-specific coding sequences of *Am*ABCC1, AmABCC2 and *Am*GSTU2a respectively (**Supplementary Table S2**) and used to generate specific antisera in rabbits, that were quality tested by ELISA (Agrisera, Vännäs, Sweden). To test the specificity of the resulting antibodies, blackgrass shoots (2-3 tiller) were homogenized in 50 mM Tris-HCl buffer p H 8.0 containing 2 mM EDTA, 250 mM sorbitol, 5 mm DTT, 0.6% (w/v) PVP (Sigma), 500 µL mL^-1^ PMSF, and 10 µL mL^-1^ of Protease inhibitor Cocktail (Sigma 9599) before centrifuging at 10,000g, 5 min, 4°C. The extract was then re-centrifuged (100,000 x g, 60 min, 4°C) to separate supernatants from microsomal pellets. Microsomal pellets were resuspended in 5 mM potassium phosphate buffer (pH 7.8) containing 330 mM saccharose, 3 mM KCL and 0.5% n-dodecyl-ß-D-maltopyranoside. After determining the protein concentration by Bradford assay (Bio-Rad, UK), 40 µg protein of the microsomal pellet (P), or supernatant (Sup), were loaded onto a 4-20% gradient gel (SDS PAGE, Bio-Rad, UK). After immunoblotting, the membrane was incubated with primary rabbit antisera raised to *Am*ABCC1, *Am*ABCC2 (dilution 1:10), or *Am*GSTU2a (1:500) overnight at 4°C. The membrane was washed with buffer before incubating with anti-rabbit HRP-conjugated secondary antibodies (Sigma-Aldrich), with immunoreactive polypeptides visualized by chemiluminescence using a ChemiDoc MP+ imaging system (Bio-Rad, UK).

### 2.7 Subcellular localisation

The full length ORFs of *AmABCC*s were codon optimised and synthesised with the restriction sites (*PacI* and *AscI;* GeneArt, Thermo Fisher Scientific) for expressing in *N. benthamiana*. The constructs were cloned into pMDC83 vector (Curtis and Grossniklaus, 2003) and the insertion confirmed by sequencing. The *Am*ABCC1-GFP and *Am*ABCC2-GFP constructs were then transformed into *Agrobacterium tumefaciens* (GV3101:PM90v).

Overnight cultures of *A. tumefaciens* containing *Am*ABCCs-GFP as prepared from single colony were diluted (1:25) in growth media containing 20 μM of acetosyringone and grown overnight. Pelleted bacteria were re-suspended in infiltration buffer (10 mM MgCL_2_, 100 μM acetosyringone) to a final OD_600_ = 0.5 and incubated at room temperature for 3 h. Cultures were mixed (1:1) with Agrobacterium containing the 35S:p19 plasmid to suppress gene silencing (Voinnet et al., 2003). For co-localisation studies, agrobacterium transformed with a plasmid encoding the specific vacuole membrane marker m-cherry (vac-rk; Nelson et al., 2007) were co-infiltrated with the *Am*ABCC-GFP constructs. The vacuole marker plasmids were obtained from the Arabidopsis Biological Resource Center (Ohio, USA). Agrobacterium were infiltrated with a needleless syringe into *N. benthamiana* leaves. Plants were kept in a growth cabinet at 21 ± 1°C at a light intensity of 120 μmol m^–2^ s^–1^, and a 16:8 h (light:dark) photoperiod for 7d. The localisation of *Am*ABCCs-GFP and marker proteins were visualised on a SP8 invert confocal microscope (Leica microsystem), with images processed and analysed using LASX software (Leica microsystem). GFP was excited at 458 nm and the signals were acquired between 468 and 558 nm. m-cherry was excited at 561 nm and acquired between 567 and 665 nm.

### 2.8 Heterologous expression of *Am*ABCC transporters in yeast

*Am*ABCC1, *Am*ABCC2 and *Am*GSTU2a coding sequences were synthesised after being codon-optimised for expression in *Saccharomyces cerevisae* (GeneArt, Thermo Fisher Scientific). For *Am*ABCC1 and *Am*ABCC2, the synthetic sequences were cloned into the NotI site of the pNEV plasmid. For *Am*GSTU2a, the sequence was cloned into the BamHI and XhoI sites of the pYES3 expression vector (Invitrogen). The yeast cadmium factor protein 1 mutant (Δ*ycf1, MAT*α *ura3-52 his6 leu2-3,-112 his3-*Δ*200 trp1-901 lys2-801 suc2-*Δ*, ycf1::hisG*), lacking the respective ABCC transporter, was transformed with pNEV or pYES3 vectors containing either *Am*ABCC1, *Am*ABCC2 or *Am*GSTU2a coding sequences to generate pNEV-*Am*ABCC1, pNEV-*Am*ABCC2 and pYES3-*Am*GSTU2a respectively. Plasmids without insertions were transformed into Δ*ycf1* and used as vector controls for all experiments.

For co- expression studies with the *Am*ABCCs and *Am*GSTU2a, yeast cells were transformed with pNEV and pYES3 empty vectors (pNEV + pYES3), or vectors containing *Am*GSTU2a in combination with either *Am*ABCC (pNEV-*Am*ABCC1 + pYES3-*Am*GSTU2a, or pNEV-*Am*ABCC2 + pYES3-*Am*GSTU2). Transformed cells were selected on minimal synthetic dropout medium lacking uracil (pNEV) or tryptophan (pYES3), supplemented with 2% (w/v) glucose at 30° on a shaker (190 rpm) for 16 h. Cells were diluted into fresh media (OD_600_ = 0.15) before treatment with 40 µM CDNB, or 20 µM mesosulfuron-methyl. Culture OD_600_ was measured at 4, 6 and 24 h following treatment. The % relative growth was determined from the OD_600_ relative to cells grown in media alone. The *ycf1* yeast mutant and pNEV vector were kindly provided by Francisco RM, University of Zurich, Institute of Plant Biology, Switzerland.

### 2.9 Statistical analysis

**Figure 2:**
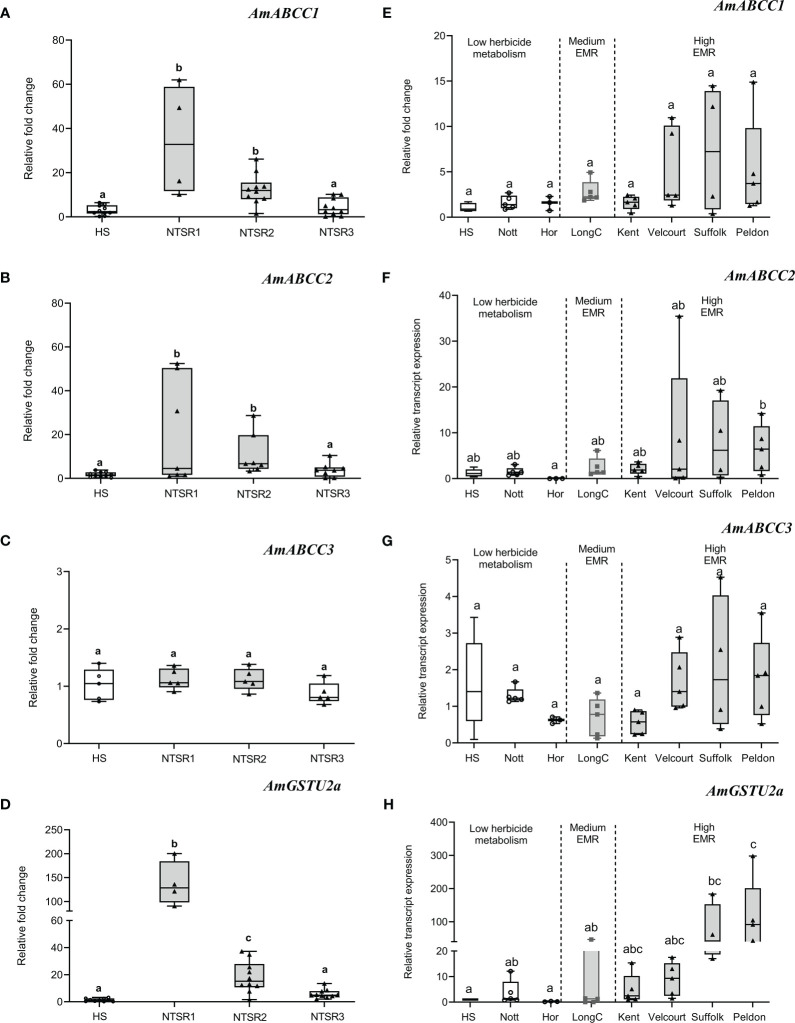
Basal relative transcript expression of *AmABCCs* and *AmGSTU2a* in NTSR and HS blackgrass populations. **(A–D)** The expression of *AmABCC1*, *AmABCC2*, *AmABCC3* and *AmGTSU2a* were quantified by qRT-PCR in HS and three NTSR blackgrass populations exhibiting the three subtypes of NTSR (Tétard-Jones et al., 2018). Box plots represent relative transcript expression (n=5) of each gene in HS, NTSR1. NTSR2 and NTSR3 populations. **(E–H)** Box plots represent the expression of *AmABCCs* and *AmGTSU2a* (n=3-5) in field-derived blackgrass populations of defined EMR phenotypes. The different letter represent statistically difference among blackgrass populations.

**Figure 3:**
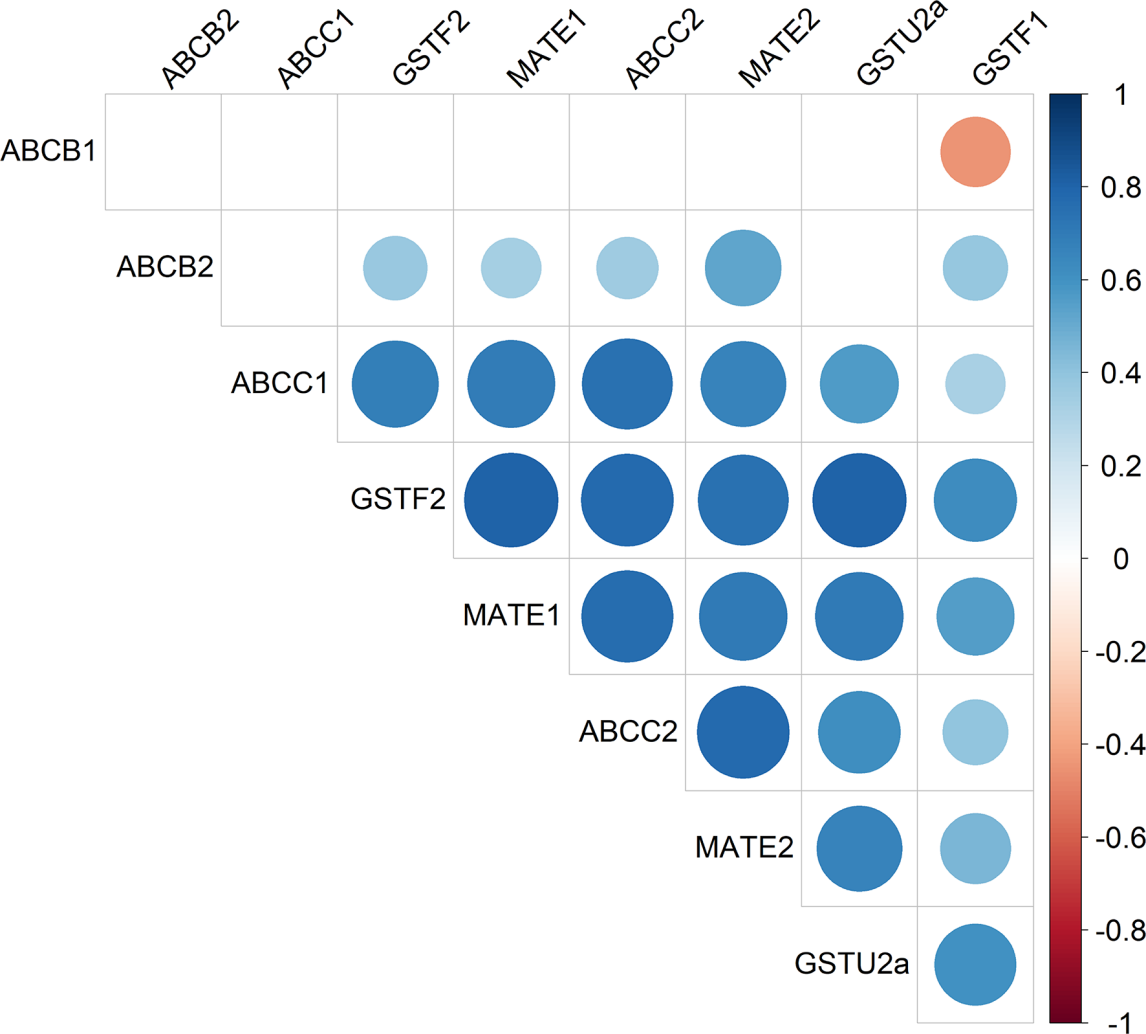
Heatmap of *AmABCC1* and *AmABCC2* transcripts as co-expressed with other genes encoding detoxification enzymes and transporters in blackgrass. The relative transcript expression of *AmABCC1, AmACC2, AmABCB1, AmABCB2, AmMATE1, AmMATE2, AmGSTU2a, AmGSTF2*, and *AmGSTF1* (n = 5) from in-field derived blackgrass populations of defined EMR phenotypes were compared by Spearman’s correlation analysis. The scale represents Spearman’s correlation coefficient, with blue circles denoting positive correlations and red circles negative correlations.

**Figure 4:**
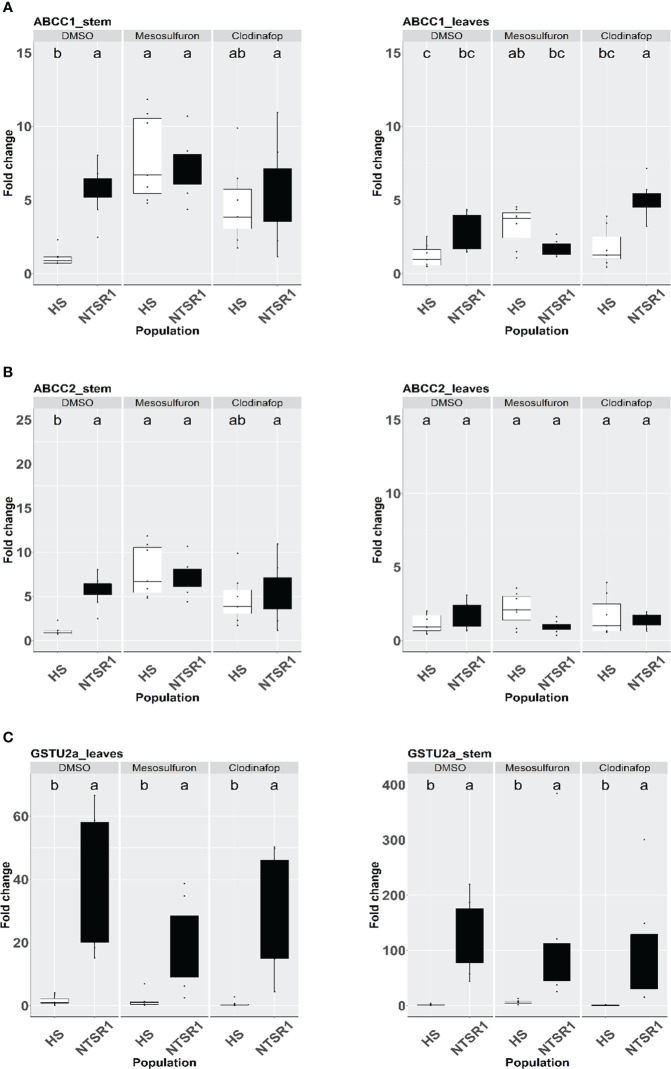
Effect of herbicide treatment on *AmABCCs* and *A*m*GSTU2a* transcript expression in HS and NTSR1 black-grass. The relative transcript expression of *Am*ABCC1 **(A)**, *AmABCC-2* **(B)**, *AmABCC3* **(C)** and *AmGSTU2a* **(D)** were determined in leaves and stems of HS and NTSR1 plants after a 24 h exposure to 40 µM mesosulfuron-methyl or 85 µM clodinafop-propargyl. Box plots represent relative expression (n=7) of transcripts in each biotype as relative fold‐expression of genes compared with solvent control treatments. Different letters designate statistical difference of transcript expression within each population.

**Figure 5:**
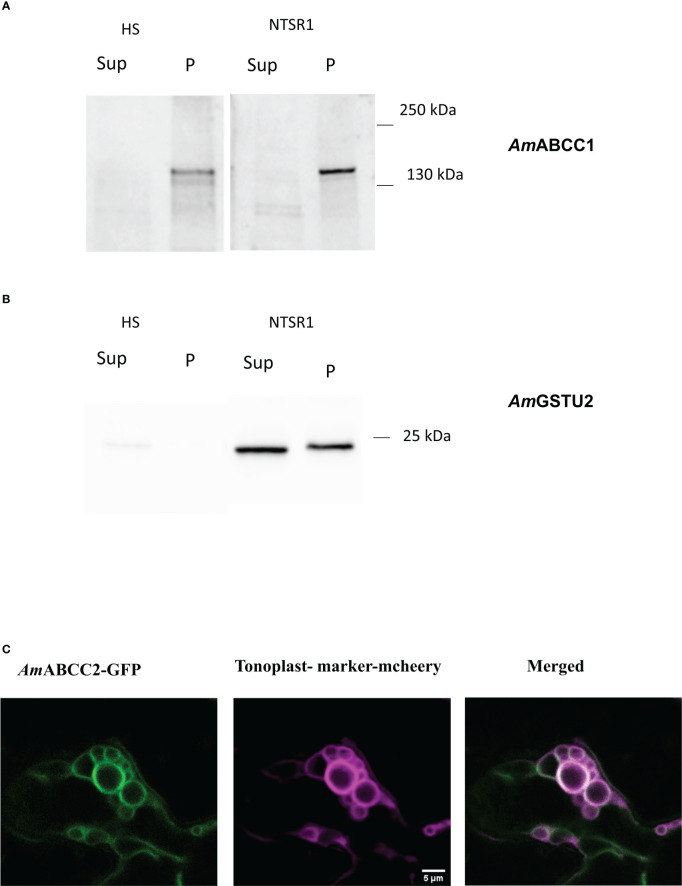
Expression of *Am*ABCC proteins in *planta*. Protein immunoblots of **(A)** *Am*ABCC1 and **(B)** *Am*GSTU2a in the soluble (Sup) and membrane fraction (P) from HS and NTSR1 blackgrass plants. **(C)** Confocal images showing intracellular localization of C-terminally GFP-tagged *Am*ABCC2 transiently expressed in *N. benthamiana* leaf epidermal cells (green). The signal of the tonoplast marker γ-TIP-RFP is shown in magenta, with the merged image showing the co-localization of *Am*ABCC2-GFP and the tonoplast marker in tobacco leaves.

**Figure 6:**
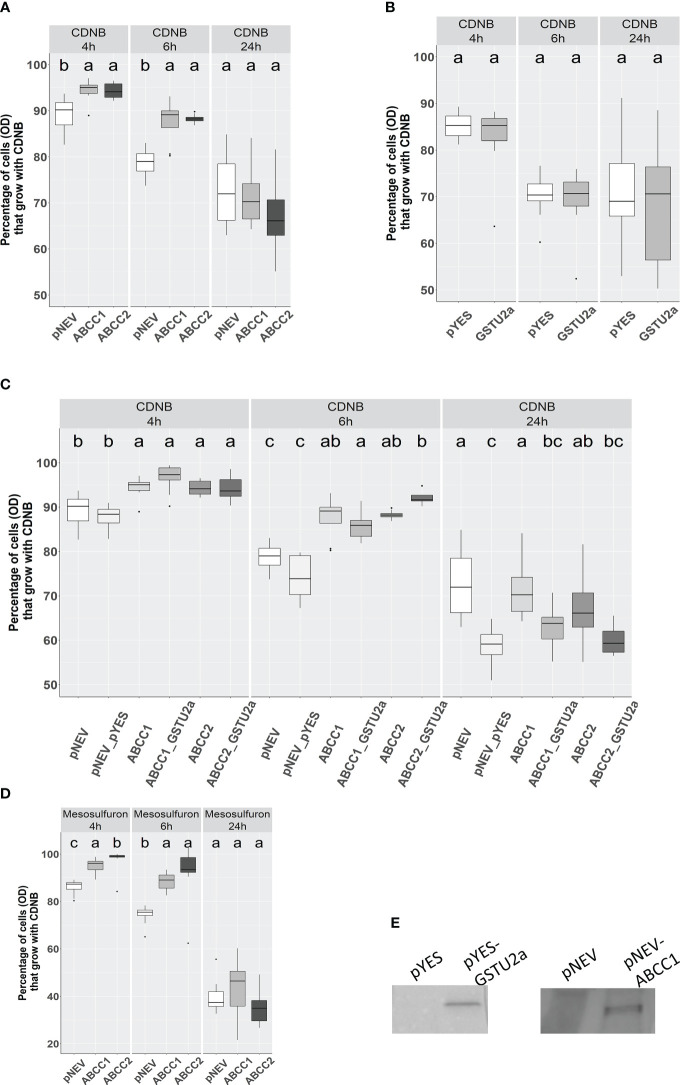
Effect of heterologous expression of *Am*ABCC1 or *Am*ABCC2 in yeast cells on tolerance to CDNB and mesosulfuron-methyl. **(A)** Effect of CDNB on *Δycf1* yeast cells transformed with either empty vector (pNEV), *Am*ABCC1, or **(B)** *Am*ABCC2. **(C)** Growth of native yeast transformed with empty vector (pYES), *Am*GSTU2a, or transformed with a combination of both vectors (pNEV + pYES) encoding each transporter along with *Am*GSTU2a. **(D)**.Effect of mesosulfuron-methyl on the growth of *Δycf1* yeast transformed with either empty vector (pNEV), *Am*ABCC1 or *Am*ABCC. **(E)** Cell extracts of yeast transformed with either empty vector (pNEV or pYES), or the respective vector containing *Am*ABCC1 or *Am*GSTU2 immunoblotted with antibodies raised against *Am*ABCC1 or *Am*GSTU. In each case, cells were grown to an OD_600_ = 0.15 and then either left untreated, or exposed to 40μM CDNB, or 20 μM mesosulfuron-methyl. The OD was then recorded at 4, h, 6 h and 24 h post treatment. Data was obtained from three independent experiments with results expressed as % growth compared to untreated cultures. The different letter represent statistically difference among blackgrass populations.

For transcript expression in **Figures 2A–D**, the data were analysed by analysis of variance (ANOVA) followed by Tukey HSD *posthoc* test using SPSS 27 (IBM, Chicago, IL, USA). For transcript expression in **Figures 2E–H**, the data were analyzed by analysis of variance (ANOVA) followed by Tukey HSD *posthoc* using R Studio. For **Figure 3**, the spearman correlations of transcript expression were analysed by R packages (corrplot and ComplexHeatmap). For **Figures 4**, **5**, **6**, the data were analyzed by one-way ANOVA followed by Tukey’s honestly significant difference (HSD) *post-hoc* test or Dunn test rstatix, FSA, multcom and rcompanion using R-Studio.

## 3 Results

### 3.1 NTSR in blackgrass is associated with the enhanced transport of conjugated xenobiotics

Enhanced translocation of conjugated xenobiotics into the vacuole by ABC and other transporter proteins is known to protect plant cells from the over-accumulation of toxic compounds in the cytosol (Hwang et al., 2016). As a primary investigation, we therefore investigated the vacuolar import of the model xenobiotic monochlorobimane (MCB) in NTSR, as compared with HS blackgrass. MCB is commonly used for studying the translocation and compartmentalisation of its fluorescent glutathionylated bimane (GS-B) conjugate *in vivo* (Coleman et al., 1997; Meyer and Fricker, 2002). GS-B is generated through the action of GSTs, with the conjugate serving as a ligand for ABC transporter proteins for vacuolar import (Coleman et al., 1997). As the accumulation of GS-B in the cytosol reduces the formation of further conjugates due to product inhibition of the GSTs, the formation and active translocation of GS-B can be monitored dynamically *in vivo* by fluorescence microscopy (Meyer and Fricker, 2002).

**Figure 7:**
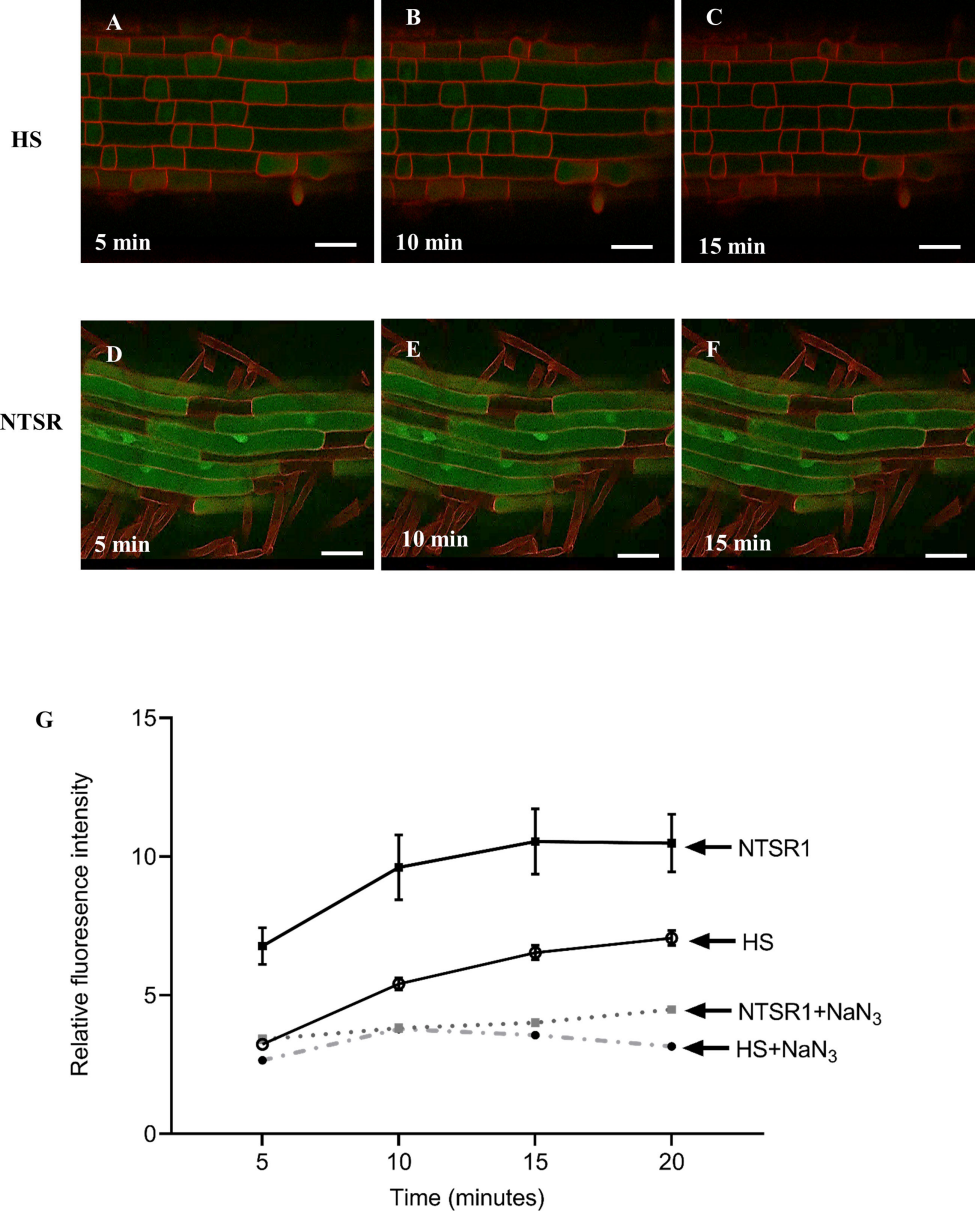
Confocal fluorescent images and intensities derived from NTSR and HS blackgrass roots treated with monochlorobimane (MCB). Confocal images from the roots of HS **(A–C)** and NTSR **(D–F)** blackgrass plants, 5, 10 and 15 min after treatment with 100 µM MCB. The fluorescent intensity of glutathione conjugated bimane (GS-B) was quantified in 20 individual cells at each time point and plotted as mean ± SD (n=20) of fluorescence intensity at each time point **(G)** for NTSR and HS blackgrass roots.

Primary roots of HS and NTSR blackgrass seedlings were exposed to 100 µM MCB and the formation of fluorescent GS-B metabolites spatially monitored and quantified using a confocal microscope over a 20 min time course (**Figures 7A–F**). The average fluorescent intensity in NTSR roots were significantly higher than those seen in the respective HS cells at all time points (**Figure 7G**; Student’s *t*-test, *P*_(5minutes)_ = 0.001, *P*_(10minutes)_ = 0.001, *P*_(15minutes)_ = 0.001, *P*_(20minutes)_ = 0.002). To investigate if the formation of the GS-B fluorescent signal was energy-dependent as would be anticipated if the action of ABC transporters were involved, sodium azide (NaN_3_), an inhibitor of cytochrome c oxidase that suppresses ATP generation, was added in combination with MCB to HS and NTSR roots. A strong reduction of fluorescent signal was observed in both HS and NTSR roots in the presence of NaN_3_, suggesting that active transport was indeed involved in GS-B accumulation (**Figure 7G**). Analysis of the images showed the greatest intensity of the signal in the NTSR root cells was associated with highly fluorescent bodies within the cells, suggestive of compartmentalisation. In contrast, the GS-B fluorescence in the HS root cells was lower at every time point than those in NTSR cells suggesting the feedback inhibition preventing further conjugation occurring, presumably due to the over accumulation of the conjugate in the cytosol. Overall, these results were consistent with NTSR blackgrass having an enhanced capacity to metabolise MCB to its fluorescent conjugate GS-B and accumulate the metabolite *in vivo* as compared with HS plants and that this process was energy dependent as a consequence of the associated ABC transporter activity.

### 3.2 Identification of ABC transporters in NTSR blackgrass

Global transcript expression analyses of NTSR and HS blackgrass populations obtained previously (Tétard-Jones et al., 2018), were analysed for sequences derived from ABC proteins, as well as from other transporter families linked to xenobiotic transport. On the basis of their relative abundance, three contigs derived from ABC transporter subfamily C genes were found to be constitutively up-regulated in NTSR, as compared with HS, blackgrass populations (**Table 1**). In addition, two members of ABC subfamily B (*AmABCB*1 and *AmABCB2*) and two (MATE) transporters, termed *AmMATE1* and *AmMATE2* were also identified (**Table 1**).

**Table 1:** Identification of contigs encoding ABC and MATE transporter proteins in the global transcriptomes of non-target site resistance (NTSR) and herbicide sensitive (HS) blackgrass **(** Tétard-Jones et al., 2018).

Gene	Transcript fold-change	NTSR vs HS
***AmABCC1***	3.1	Upregulated in NTSR
***AmABCC2***	3.1	Upregulated in NTSR
***AmABCC3***	3.6	Upregulated in NTSR
***AmABCB1***	6.9	Upregulated in HS
***AmABCB2***	2.6	Upregulated in HS
***AmMATE_1***	14.1	Upregulated in NTSR
***AmMATE_2***	12.9	Upregulated in NTSR

**Figure 8:**
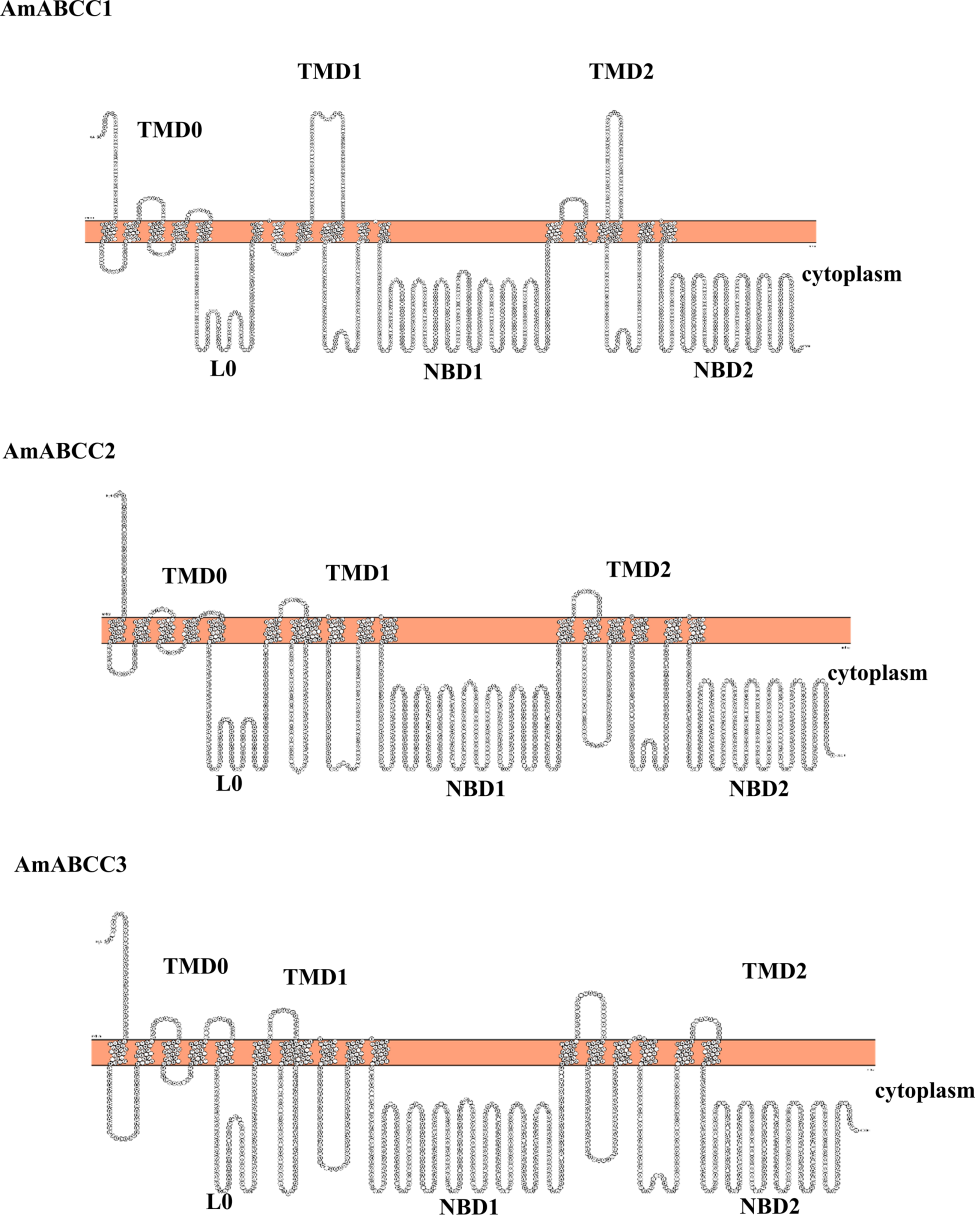
Predicted topology of *Am*ABC sub-family C transporters. The topology models of *Am*ABCC1, *Am*ABCC2 and *Am*ABCC3 membrane proteins illustrating the two cytoplasmic nucleotide-binding domains (NBDs) and the three transmembrane domains (TMDs).

Based on their relative enhanced expression in NTSR plants and sequence coverage, it was possible to assemble full-length DNA sequences representing distinct ABCC transporter proteins. The sequences of the assembled ORFs were then confirmed following their RT-PCR amplification from NTSR plants. To further confirm their identity, the full-length sequences were subjected to a web server analysis (TOPCONS) for topology prediction. All three *Am*ABCC sequences had an orientation TMD-NBD-TMD-NBD with an additional N terminal transmembrane domain (TMD0) and a cytosolic linker region (L0) (**Figure 8**), typical of the ABC-C subfamily (Biemans-Oldehinkel et al., 2006). The sequences of NBDs exhibited the typical motif for ATP binding and hydrolysis, termed the Walker A and B motifs respectively (Walker et al., 1982; **Supplementary Figure S1**). With this topology analysis confirming the three *Am*ABCs belonged to subfamily C, they were subsequently named as *Am*ABCC1, *Am*ABCC2 and *Am*ABCC3 respectively. It is noteworthy that the *in-silico* analysis using WolF PSORT and SignalP tools could not detect the sequence that could pinpoint the localization of the three transporters.

### 3.3 Phylogenetic analysis of the three blackgrass ABCC transporters

**Figure 9:**
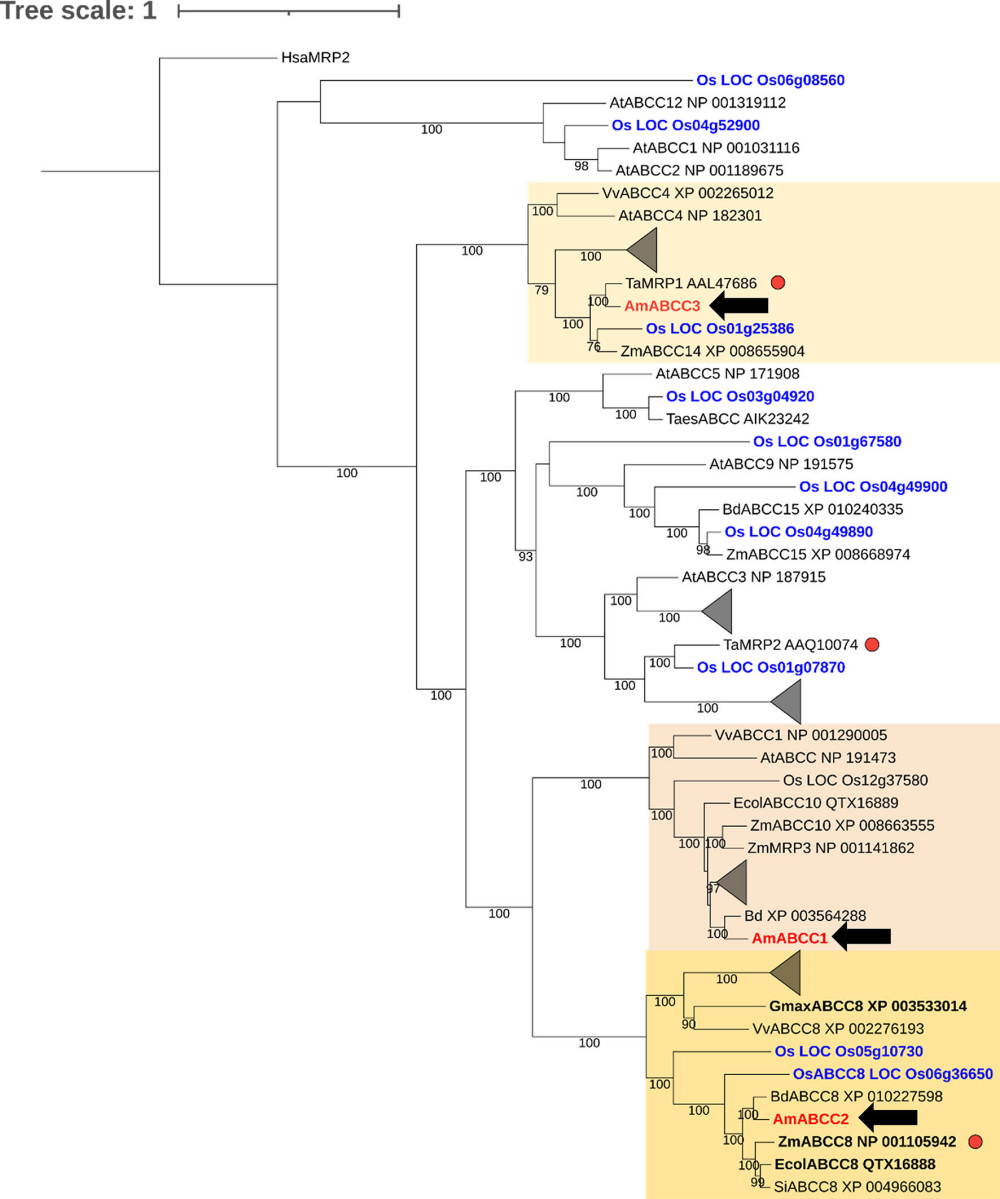
Phylogenetic analysis of ABCC proteins in grass weeds and crop plants. Amino acid sequences of *Am*ABCC1, *Am*ABCC2, and *Am*ABCC3 from blackgrass *(Am*, *Alopecurus myosuroides*; red), rice *(Os*, *Oryza sativa*; blue) wheat *(Ta*, *Triticum aestivum*), maize (*Zm*, *Zea mays*), Arabidopsis (*At*, *Arabidopsis thaliana*), grape vine (*Vv*, *Vitis vinifera*), purple false brome (*Bd*, *Brachypodium distachyon)*, jungle rice (*Ec*, *Echinochloa colona*), and foxtail millet (*Si*, *Setaria italica*) were used for maximum likelihood alignment for phylogenic analysis. The number on the branch represents the bootstrap support values above 75%. The scale bar indicates the inferred number of substitutions per site. Clades comprising exclusively rice and Arabidopsis sequences were collapsed into triangles. Sequences in bold correspond to proteins that confer resistance to glyphosate when enhanced *in planta* (Pan et al., 2021). Red dots indicate genes induced by safeners. The tree was rooted using the sequence of human MPR2 (*Hsa, homo sapiens* MRP2).

ABCC transporters are known to transport plant metabolites, including conjugated xenobiotics in several plant species (Bartholomew et al., 2002; Goodman et al., 2004; Francisco et al., 2013; Behrens et al., 2019). To explore the prospective functions of the newly identified *Am*ABCCs in blackgrass, a phylogenetic analysis was conducted with the ABC sequences identified in black-grass and ABC homologues. The tree was constructed with all the annotated ABCC transporter sequences present in rice (Verrier et al., 2008). The three *Am*ABCCs shared between 37% and 41% similarity in amino acid sequences to each other, with each clustered in distinct clades (**Figure 9**)

*Am*ABCC1 clustered with grape ABCC1 (*Vitis vinifera*, *Vv*ABCC1) and maize MRP3 (*Zea may*; *Zm*MRP3). Both transporters have been reported to function as anthocyanin transporters (Goodman et al., 2004; Francisco et al., 2013). *Am*ABCC2 formed a distinct monophyletic group with ABC proteins collectively annotated as ABCC8 transporters, comprising *Ec*ABCC8 (*Echinochloa colona*), *Gm*ABCC8 (*Glycine max*; soybean), *Zm*ABCC8 (*Zea mays)* and *Os*ABCC8 (*Oryza sativa*; rice). While the role of these transporters in endogenous plant metabolism is unknown, *Ec*ABCC8 has been shown to be responsible for contributing to glyphosate resistance in jungle rice (Pan et al., 2021). The related *Zm*ABCC8 shared 98% amino acid similarity with *Zm*MRP1, a transporter protein induced by safeners that enhance herbicide detoxification in maize (Pang et al., 2012). Based on the high similarity of amino acid sequences between *Zm*ABCC8 and *Zm*MPR1, these two proteins mapped to the same position in the phylogenetic tree. Therefore, ZmMRP1 was not shown in the tree. With respect to *Am*ABCC3, this protein clustered with a largely uncharacterised transporter from wheat (*Triticum aestivum*) termed *Ta*MRP1, which is induced following herbicide safener treatment (Theodoulou et al., 2003). This phylogenetic analysis suggested the prospective biological functions of ABCCs. Whereas *Am*ABCC1 probably functions in endogenous metabolite transportation, *Am*ABCC2 and *Am*ABCC3 could potentially be involved in EMR based on the sequence similarity to transporters in other plant species linked to tolerance to xenobiotics and herbicides.

### 3.4 Expression of the three ABCC transporters in different NTSR blackgrass populations

Over the course of multiple studies, the molecular basis of the NTSR phenotype has been partially defined in multiple blackgrass populations (Tétard-Jones et al., 2018; Franco-Ortega et al., 2021). Based on this analysis, three major types of NTSR have been described; NTSR1 derived in the field following exposure to multiple classes of herbicides; NTSR2 which arises when HS plants are repeatedly selected for tolerance to the herbicide pendimethalin and NTSR3, arising from selection using fenoxaprop-ethyl (Tétard-Jones et al., 2018; Franco-Ortega et al., 2021). While NTSR1 and NTSR2 populations exhibit a classic upregulation of detoxification enzymes consistent with EMR, NTSR3 plants are resistant due to a distinct mechanism that does not involve enhanced herbicide metabolism. To test for the association between enhanced expression of the different ABCC genes and the different NTSR traits, the transcript expression of each set of plants were subjected to qRT-PCR analysis to quantify the relative abundance of each transporter relative to an HS population (**Figure 2**). For reference, the gene encoding the tau (U) class glutathione transferase *AmGSTU2a* was included, as the respective enzyme is highly active in detoxifying chloroacetanilide herbicides and showed a high level of correlation with the degree of EMR found in different blackgrass populations (Nandula et al., 2019). The relative expressions of *AmABCC1* and *AmABCC2* were found to be comparable in the NTSR1 and NTSR2 populations (**Figures 2A, B**; one-way ANOVA; *P_(AmABCC1_)* = 0.99; *P_(AmABCC2_)* = 0.98) and significantly higher than those determined in HS or NTSR3 plants (**Figures 2A**, **9B**; one-way ANOVA; *P* < 0.05). In contrast, *AmABCC3* relative expression was similar in all the blackgrass populations tested (**Figure 2C**, one-way ANOVA; p > 0.05). This finding suggested that *AmABCC1* and *AmABCC2* were more likely to link to EMR than *AmABCC3*. It is intriguing that while phylogenetic analysis showed that *AmABCC3* was related to ABCCs in other species that function in xenobiotic detoxification, the transcript expression of AmABCC3 were unaltered in NTSR blackgrass populations. This discrepancy prompted us to further confirmed the expression of *AmABCCs* in wider blackgrass populations.

To further assess quantitative links between levels of the expression of the *AmABCC* genes and the degree of resistance conferred by enhanced herbicide metabolism, a further seven field derived NTSR blackgrass populations were tested that had previously been characterised with respect to the relative degrees of EMR exhibited (Marshall et al., 2013; Sabbadin et al., 2017). While the ‘Kent’, ‘Velcourt’, ‘Suffolk’ and ‘Peldon’ populations show high levels of EMR, ‘LongC’ was classified as possessing medium EMR, with ‘Notts’ and ‘Hor’ primarily resistant due to TSR mechanisms alone (Sabbadin et al., 2017). Due to the genetic variation within these field-derived populations, the expression of each of the *AmABCCs* showed high degree of variation within population which lead to the non-normal distribution of transcript expression data. Using non-parametric analysis, levels of expression of all three *AmABCC* genes were not statistically different among the TSR populations (Nots, Hor) the HS and EMR populations (Kent, Velcourt, Suffolk, Peldon and LongC). However, the expression trend of *AmABCC1* was shown to be elevated in the highly-EMR populations Velcourt, Suffolk and Peldon, while no enhancement was determined in Kent (high) or LongC (medium) relative to that determined in HS blackgrass (**Figure 2E**). In the case of *AmABCC2*, expression tended to increase in all EMR populations (**Figure 2F**), while the transcript abundance of *AmABCC3* was comparable in all plants irrespective of their resistance status (**Figure 2G**).

As a point of reference, the studies of *AmGSTU2a* broadly demonstrated the validity of linking the transcript abundance of key detoxification genes with the degree of EMR exhibited (**Figures 2D, H**). With respect to the transporters, collectively these results were consistent with *AmABCC*1 *and AmABCC2* being linked to EMR in herbicide resistant blackgrass. In contrast, the similar levels of expression of *AmABCC3* observed in the blackgrass populations irrespective of their resistance status suggested that this transporter was not linked to NTSR or EMR. These results highlight the diversity of functions of ABCCs transporters among plant species that could not be predicted based on sequence similarity. Based on the results of these gene expression studies, the further analysis of the transporters and their role in EMR was focused on *AmABCC1* and *AmABCC2*.

### 3.5 Co-expression of *Am*ABCC1 and *Am*ABCC2 with other genes linked to herbicide detoxification and EMR

Our previous study reported the enhanced co-expression of genes encoding proteins with linked metabolic functions in NTSR blackgrass including those involved in herbicide detoxification (Tétard-Jones et al., 2018). As such, it was determined whether the transporters *Am*ABCC1 and *Am*ABCC2 were co-expressed with the genes encoding other transporters and detoxifying enzymes in the transcriptomes of different NTSR blackgrass populations (Tétard-Jones et al., 2018). The genes selected for correlative analysis included the ABC transporters from family B (*AmABCB1*, *AmABCB2*), the *AmMATE1* and *AmMATE2* transporters, the (phi F) glutathione transferases *AmGSTF1*, *AmGSTF2* as well as *AmGSTU2a.*

Relative gene expression was determined by qRT-PCR, with *AmABCC1* and *AmBCC2* subjected to correlation analysis with the other six genes present in the meristem tissues of the seven field-derived herbicide resistant blackgrass populations. Analysis confirmed the positive correlations in the expression of *AmGSTU2a* with *AmABCC1* (Spearman’s correlation*, r_(37)_* = 0.56, p < 0.05), and with *AmGSTU2a* and *AmABCC2* (*r_(37)_* = 0.62; p < 0.05) (**Figure 3**). Besides *AmGSTU2a*, the relative expressions of *AmABCC1*and *AmABCC*2 also positively correlated with *AmGSTF2* and *AmMATE2* (**Figure 3**; Spearman’s correlation, *r*_(A_ *_m_*_ABCC1-_ *_Am_*_GSTF2)_ = 0.68, *r*_(_ *_AmABCC2_*_-AmGSTF2)_ = 0.77, *r* = _(_ *_AmABCC1- AmMATE2_*_)_ = 0.70, *r* = _(_ *_AmABCC2_*_-_ *_AmMATE2_*_)_ = 0.77; p = < 0.05). *AmGSTF1* was also included in the correlation analysis as a previously defined biomarker of NTSR in blackgrass (Tétard-Jones et al., 2018; Comont et al., 2020). As anticipated, *AmGSTF1* transcript expression was higher in NTSR/EMR populations (**Figure S2**) and found to positively correlate with *AmGSTU2a* (**Figure 3**; Spearman’s correlation; *r_(37)_* = 0.61, *P* < 0.05) and *AmABCC2* (**Figure 2**; Spearman’s correlation; *r_(37)_* = 0.38, *P* = 0.19). The positive correlation in the expression of the two *AmABCCs* and *AmGSTU2a* were particularly interesting, being consistent with coupled functions of a herbicide conjugating enzyme and transporters capable of rapidly removing conjugation products in EMR plants. In contrast, the correlation between *AmGSTF1* and the *AmABCC* transporters was weaker than that determined with *AmGSTU2a*, suggesting any coupling in function was less direct.

### 3.6 Regulation of *Am*ABCC transporters by exposure to herbicides

Genes involved in xenobiotic detoxification are often induced when plants are exposed to chemical injury (Xu et al., 2015). As such it was of interest to determine if the *AmABCC*s and the functionally linked *AmGSTU2a* which were all constitutively highly expressed in EMR blackgrass, were responsive to chemical treatments. In the first instance, HS and NTSR1 (Peldon) plants were exposed to two herbicides with differing chemistries and modes of action, namely the sulfonylurea mesosulfuron-methyl and the aryloxyphenoxypropionate clodinafop-propagyl. These two herbicides were selected since they had been widely used to control blackgrass and are both subject to evolved NTSR in the field. With each treatment, the relative expression of *Am*A*BCC1*, *AmABCC2* and *AmGSTU2a* were determined in the leaf and meristem tissues of HS and NTSR1 plants 24h after foliage application of herbicides, or solvent control.

*AmABCC1, AmABC2 and AmGSTU2a* expression were significantly higher in NTSR blackgrass compared to those determined in HS blackgrass at the constitutive level (untreated plants). The significantly higher expression of these genes in NTSR (Peldon) blackgrass were also observed in the solvent control treatment (**Figures 4A–C**). In the HS plants, the 24 h exposure to mesosulfuron resulted in a major induction of *AmABCC1* and *AmABCC2* in the meristems, comparable to that determined in NTSR plants, but not in the leaves (**Figures 4A, B**; one-way ANOVA; p < 0.05). This enhancement in response to herbicide treatment was not observed in any tissue in the HS plants with the *AmGSTU2a* gene (**Figure 4C**). It is noteworthy that while herbicide treatment did not enhance the expression of *AmABCCs* or *AmGSTU2*a in meristems of NTSR plants, clodinafop treatment significantly induced the expression of *AmABCC1* transcripts in NTSR plants (**Figures 4A–C**).

### 3.7 Expression of *Am*ABCC proteins *in planta*

To study the expression of *Am*ABCC1 and *Am*ABCC2 proteins in blackgrass plants, polyclonal antibodies were generated to peptide sequences specific to each transporter. In addition, an antibody was raised against native *Am*GSTU2a (Nandula et al., 2019). All three antisera were then used to probe a membrane protein (P) and a total soluble protein (Sup) fraction derived from meristem extracts from HS and NTSR1 blackgrass. After separating by SDS-PAGE, the polypeptides present in the two fractions were blotted and tested with the respective antisera. The anti-*Am*GSTU2a-sera readily detected polypeptides with a relative molecular mass (MW) 24.5 kDa, which is typical for GSTU subunits, in the soluble fraction (Sup) in NTSR1 plants (**Figure 5B**). A polypeptide with a slightly higher MW was also detected with this antisera in the membrane fraction (P). The peptide antisera raised against *Am*ABCC1 detected a polypeptide with a relative molecular mass ~150kDa, consistent with it being derived from an ABC protein, in the membrane fraction (P) of HS and NTSR 1 blackgrass (**Figure 5A**). Densitometric analysis showed that the signal associated with the band from NTSR1 plants was double that determined in the extracts from HS plants. In contrast, it was not possible to detect membrane or soluble polypeptides using the *Am*ABCC2 antisera in these preparations, even though the antibody had been demonstrated to recognise its targeted peptide sequence with equal affinity to that raised against *Am*ABCC1 in earlier ELISA tests. From this it was concluded that at the level of expressed protein, *Am*ABCC2 is less abundant than *Am*ABCC1 in NTSR blackgrass.

The subcellular localisation of the *Am*ABCC proteins *in planta*, was studied by transiently-expressing each transporter C-terminally fused with green fluorescent protein (GFP) under the control of the cauliflower mosaic virus 35S promoter in the leaves of *Nicotiana benthamiana*. Both *Am*ABCC-GFPs were co-expressed with the aquaporin tonoplast marker γ-TIP fused with m-cherry (Nelson et al., 2007). The resulting localisation of the fluorescent fusion proteins was then monitored by confocal microscopy. The signal from *Am*ABCC2-GFP co-localised with the tonoplast marker in small vesiculated bodies (**Figure 5C**). However, the fluorescent signal from *Am*ABCC1-GFP could not be detected, even though the signal from the tonoplast marker was clearly visible in the sample (**Figure S3**). The localization of *Am*ABCC2 in the tonoplasts of blackgrass were consistent with a role of actively importing transport toxic compounds into vacuoles as determined for related ABC transporters in other plant species (Liu et al., 2001; Jaquinod et al., 2007). It is noteworthy that while we could not confirm the localization of *Am*ABCC1 in the tonoplast using a transient expression approach, the subcellular fractionation of the protein in blackgrass plants confirmed its association with membranes.

### 3.8 Effect of heterologous expression of *Am*ABCC1 and *Am*ABCC2 on xenobiotic tolerance in transgenic yeast

To investigate any cytoprotective effect of these transporters towards xenobiotics and herbicides, *Am*ABCC1 and *Am*ABCC2 were expressed in *Saccharomyces cerevisiae* Δ*ycf1*strain defective in the cadmium factor protein, an ABCC transporter. These Δ*ycf1* cells are susceptible to xenobiotics including the model GST substrate 1-chloro-2,4-dinitrobenzene (CDNB), in a dose responsive manner (Li et al., 1996). As such, the protective effect of plant ABC transporters in removing toxic xenobiotics from the yeast cytosol for deposition in the vacuole, or extracellular transport can be functionally assessed following their heterologous expression in the Δ*ycf1* mutants through relative growth assays.

Yeast Δ*ycf1* cells were transformed with either *Am*ABCC1 or *Am*ABCC2 and along with vector only controls (pNEV), exposed to 40 µM CDNB and the effect on growth determined by densitometry over 24 h as compared with untreated cultures, whose cell density were taken as being 100% at each time point. The percentage OD600 in cultures expressing either *Am*ABCC1, or *Am*ABCC2, were significantly higher than the pNEV controls at 4 h and 6 h after treatment (one-way ANOVA, p < 0.05; **Figure 6A**). By 24 h this protective was no longer seen with all the cultures showing around 30% growth inhibition.

CDNB undergoes *S*-glutathionylation in cells containing the tripeptide glutathione through a combination of spontaneous conjugation and GST-catalysed reactions. The positive correlation in the co-expression of both *Am*ABCCs with *Am*GSTU2a in NTSR blackgrass (**Figure 3**), and the inherently high activity of *Am*GSTU2a toward CDNB (Nandula et al., 2019), prompted us to test whether co-expression of this GST with the transporters could enhance the protection to CDNB by providing a catalyst for detoxifying this xenobiotic. It was then postulated that the resulting CDNB conjugates would be removed from the cytosol by the *Am*ABCCs thereby accelerating the overall rates of detoxification. The CDNB toxicity trial was therefore re-run with *Am*GSTU2 co-expressed with the *Am*ABCC transporters along with the vector only control and expression of the respective proteins (*Am*ABCC1 and AmGSTU2) confirmed by immunoblotting with the respective antisera (**Figure 6E**). The results obtained showed no additive protective effect of co-expressing the GST with either transporter (**Figure 6C**). In addition, expression of *Am*GSTU2a alone also showed no protective effect toward CDNB (**Figure 6B**), though it was not possible to confirm that the transformed cells were accumulating the respective conjugate. Taken together these results suggested that the activity of the transporter alone was able to protect the Δ*ycf1* cells, though it was unproven as to whether the *Am*ABCCs were translocating the native xenobiotic, or its detoxification products.

To examine the protective effect of the transporters in the yeast assays in further detail the growth of the *Am*ABCC-transformed Δ*ycf1* cells were determined in the presence of 8 different herbicide chemistries used to control blackgrass, including pendimethalin, fenoxaprop-ethyl, mesosulfuron-methyl and glyphosate. Of the compounds tested only the sulphonyl urea mesosulfuron-methyl, inhibited the growth of Δ*ycf1* cell growth (**Figure S4**). This inhibitory effect was markedly reduced in the cells expressing *Am*ABCC1 and *Am*ABCC2 at the 4 h and 6 h timepoints, though it was not apparent at 24 h, potentially as a consequence of the cultures entering the stationary phase (**Figure 6D**).

## 4 Discussion

Using a combination of transcriptomics, protein expression studies *in planta* and functional characterisation studies in transgenic yeast, two ABCC transporters were found to be linked to EMR toward multiple herbicides in the problem weed blackgrass. While recognizing that a fully definitive link between herbicide resistance and the functional expression of these transporters would need exhaustive forward and reverse genetic studies in the future, our studies provide further evidence of the importance of the biotransformation and intracellular deposition of herbicides and their metabolites in NTSR weeds. Despite the known importance of ABC transporters in plant metabolism, relatively few studies have led to their functional characterization, in part because of the technical difficulties in studying these integral membrane proteins. In the case of natural product metabolism, ABC transporters have been shown to function in the vacuolar import of flavonoids and anthocyanins (Francisco et al., 2013), while others are located in the plasma membrane and involved in cuticle and pollen coat deposition (Choi et al., 2014). These transporters have also been shown to have a protective role in the detoxification of pollutants and herbicides (Lu et al., 1997; Lu et al., 1998), through a combination of vacuolar deposition as well as extra-cellular efflux (Park et al., 2011; Pan et al., 2021). The current studies confirm that *Am*ABCC2 localized to the tonoplast membrane and is therefore most likely involved in vacuolar import. While its association with membranes was determined, the subcellular localization of AmABCC1 could not be confirmed by transient expression in Nicotiana, though we speculate that this protein would associate with the plasma-membrane.

While the ABC family in plants is large, it is interesting in blackgrass, that only two members of the C family appear to be linked to EMR-mediated herbicide resistance. Similarly, plants contain a large family of membrane bound Cytochromes P450 monooxygenases, yet only a very small number of them derived from the CYP81 family are important in herbicide metabolism in both crops and weeds (Dimaano and Iwakami, 2021). Such findings suggest that the metabolism of the large number of diverse xenobiotics encountered by plants is carried out by a small number of enzymes and transporters able to accept a broad range of synthetic substrates rather than relying on the innate diversity of the superfamilies of proteins with potential roles in biotransformation. Interestingly the ABCC proteins linked to EMR in blackgrass have orthologs in other species which have been linked to xenobiotic metabolism and/or herbicide tolerance. *Am*ABCC1 is related to the multidrug resistance–associated protein, *Zm*MRP3. *Am*ABCC2 is related to two transporters which have been linked to herbicide tolerance namely *Zm*MRP1 and *Ec*ABCC8. *Zm*MRP1 has been shown to be up-regulated following exposure to herbicides and safeners (Pang et al., 2012) which suggest its involvement in herbicide detoxification in crop species. Importantly, the relatedness of *Am*ABCC2 to *Ec*ABCC8 from jungle rice is particularly intriguing as this transporter is linked to glyphosate resistance in jungle rice (*Echinochloa colona*). While we were unable to confirm any functional role in glyphosate transport in our heterologous yeast expression system, based on phylogeny *Am*ABCC2 would be a prime candidate for such a function in blackgrass. While full-blown glyphosate resistance has not been reported in blackgrass, studies have shown that the tolerance to this critical herbicide is slowly evolving in the UK, being associated with the evolution of NTSR (Comont et al., 2019). It is therefore concerning that while evolving a role in NTSR toward currently used selective herbicides, that the associated enhanced expression of *Am*ABCC2 could ultimately lead to the unexpected development of resistance toward glyphosate in blackgrass and potentially other wild grasses.

Based on differences in their phylogeny and the known differences in function of the respective orthologs in other plants, we originally speculated that the functions of *AmA*BCC1 must differ from those of *Am*ABCC2. However, both transporters promoted very similar levels of protection to CDNB and mesosulfuron-methyl in the transgenic yeast assays. In both cases it did not prove possible to identify the nature of the transported ligands as being the parent xenobiotics, or the respective conjugated metabolites. The available evidence would suggest the protective effect was due to the efflux or vacuolar sequestration of the parent compounds. In the case of CDNB, co-expression of *Am*GSTU2a which actively *S*-glutathionylates this substrate provided no additional protection, suggesting conjugation was not critical to detoxification. In the case of mesosulfuron, while S-glutathionylation has been proposed to be a later step in the metabolism of the herbicide (Zhao et al., 2019), this reaction is unlikely to be a primary detoxification reaction. Instead, it would seem in the yeast system at least, that the *Am*ABCC transporters must be acting on the parent compounds directly, possibly using endogenous metabolites such as glutathione for co-transport, as has been postulated for anthocyanin and flavonoid translocation (Francisco et al., 2013). While the two transporters may have similar functional roles in xenobiotic detoxification, their activities toward endogenous metabolites may well differ, with *Am*ABCC1 being related to transporters known to be active in anthocyanin deposition in the vacuole. Intriguingly, enhanced anthocyanin accumulation is associated with the NTSR1 phenotype in blackgrass (Cummins et al., 2009), suggesting that as a consequence of selecting for enhanced herbicide transport, the plants also acquire an increased ability to sequester anthocyanin pigments.

The transcript expression study with the different NTSR populations of blackgrass suggested the link between the NTSR1 and NTSR2 resistance phenotype and the consistently enhanced levels of transcripts encoding *Am*ABCC1 and *Am*ABCC2 (**Figures 2A, B**). As demonstrated with a number of gene families involved in herbicide metabolism (Franco-Ortega et al., 2021), the enhancement in transcript levels was considerably greater than that determined at the protein level *in planta*, which suggests an element of post-transcriptional regulation. Such a complex level of controlling gene expression may well reflect the important roles these proteins play in multiple transport processes in the cell. As such, while it may be desirable from a herbicide-resistance selection stand point to over-express ABC transporters, this may have effects on endogenous metabolism and homeostasis that impose a fitness penalty. Consistent with such a tight level of gene regulation, while both *Am*ABCCs were responsive to herbicide exposure in the HS blackgrass, this was not the case in the NTSR plants. This suggests that once selected to constitutively display the NTSR phenotype, the *Am*ABCC genes are not further responsive to short term chemical injury.

These results further exemplify the importance of ABC transporters in resistance to xenobiotics in living organisms that encompass multidrug resistance in cancer cells (Cole et al., 1992; Robey et al., 2007), antibiotic resistance in bacteria (Greene et al., 2018), and resistance to insecticides (Park et al., 2014) and fungicides (Nakaune et al., 1998) in crop pests and pathogens. The identification of two ABCC family members as being specifically linked to EMR conferring resistance to multiple herbicides in blackgrass will be of assistance in identifying orthologs in other wild grasses linked to herbicide resistance. While controlling NTSR weeds in the field is problematic, identifying the role of specific ABC transporters may provide new druggable targets to reverse herbicide resistance as proposed to improve chemotherapy efficacy in cancer treatment (Choi, 2005). As such, future studies to identify *Am*ABCC transport ligands will provide information on new leads for synthetic or natural product-based inhibitors of these transporters to be developed as ‘resistance-busting’ herbicide synergists.

## Data availability statement

The data presented in this study are deposited in the NCBI GeneBank repository, accession number OM621837 (AmABCC1), OM621836 (AmABCC2), OM621838 (AmABCC3), OM621840 (AmMATE1), OM621839 (AmMATE2), OM621841 (AmABCB1), KY172655 (AmGSTU2a), AJ010453 (AmGSTF1).

## Author contributions

RE conceptualized, supervised and funded the research. AGC carrying out the informatics, transient expression, antisera experiments and data analysis, SF-O performed herbicide treatment experiment and data analysis, NO performed bioimaging experiments and data analysis. RE, AGC and NO wrote and edited manuscript. All authors contributed to the article and approved the submitted version.

## Supplementary material

Supplementary Table 1 Primer specific sequences for confirmation of *AmA*BCC identities and for real-time qRT-PCR analysis

Supplementary Table 2 Amino acid sequences used to generate specific antisera for *Am*ABCC1, *Am*ABCC2 and *Am*GSTU2a

Supplementary Table 3 A list of blackgrass populations used in this study. The herbicide resistance status of blackgrass populations has been confirmed by herbicide resistance assay and analysis of mutation in ALS or ACCase protein.

Supplementary Figure 1 Amino acid sequences analysis of *Am*ABCC1, *Am*ABCC2, *Am*ABCC3 and *Zm*MPR3 proteins, with the typical motifs for the ABC signature and ATP binding and hydrolysis (Walker A and B motifs) shown.

Supplementary Figure 2 The relative transcript expression of *AmGSTF1* in field-derived blackgrass populations collected across the UK with characterized EMR phenotypes. The herbicide sensitive (HS), target site mutation (Nott, Hor) and NTSR (LongC, Kent, Velcourt, Suffolk, Peldon) population were used. Box plots represent relative transcript expression (n=5).

Supplementary Figure 3 The subcellular localization of the tonoplast marker (γ-TIP) fusion with m-cheery in *Nicotiana benthamiana* leaves.

Supplementary Figure 4 The effects of herbicides on yeast cell growth. *Am*ABCC was expressed in yeast cells which were then treated with nicosulfuron, chlorotoluron, CDNB, pendimethalin, tembotrione, glyphosate, fenoxaprop-ethyl, bentazone and mesosulfuron-methyl. The OD_600_ were determined at 2, 4, 6 and 24 h after treatment with either 20 µM or 40 µM herbicides.
