# Supplementary material for: ABC transporters linked to multiple herbicide resistance in blackgrass (Alopecurus myosuroides)
Source: Front Plant Sci. 2023 Mar 17;14:1082761. doi: 10.3389/fpls.2023.1082761 (PMC10063862; doi:10.3389/fpls.2023.1082761)
Supplement: Supplementary Table 1 — Primer specific sequences for confirmation of AmABCC identities and for real-time qRT-PCR analysis [file DataSheet_1.docx]

Supplementary Material

**ABC transporters linked to multiple herbicide resistance in blackgrass (*Alopecurus myosuroides*)**

Alina Goldberg Cavalleri ^1^, Nawaporn Onkokesung^1^, Sara Franco Ortega^1$^, Robert Edwards^1*^

^1^Agriculture, School of Natural and Environmental Science, Newcastle University, Newcastle Upon Tyne, NE1 7RU, UK

^$^ Present address: University of York, Department of Biology, Heslington, York, YO10 5DD, UK

*Corresponding author: [robert.edwards@newcastle.ac.uk](about:blank)

**Supplementary Tables**

**Supplementary Table S1** Primer specific sequences for confirmation of *AmA*BCCs and for real-time qRT-PCR analysis

| Type | Gene | Sense (5’-3’) |
| --- | --- | --- |
| Sequence confirmation | *AmABCC-1-UTRF* | GCTGAAAGGGGATCCACTCGGC |
|  | *AmABCC-1-UTRR* | AGTACTCATTGACCAGATCGCG |
|  | *AmABCC-1-707F* | GATAGTAAGGTGACTCCTTTAGC |
|  | *AmABCC-1-707R* | GCTAAAGGAGTCACCTTACTATC |
|  | *AmABCC-1-1533F* | ATGCATGGGAAGCTCACTTCAAG |
|  | *AmABCC-1-1533R* | CTTGAAGTGAGCTTCCCATGCAT |
|  | *AmABCC-1-2360F* | GTTGATGCTCATACAGCGACAAG |
|  | *AmABCC-1-2360R* | CTTGTCGCTGTATGAGCATCAAC |
|  | *AmABCC-1-3189F* | GGCAAGTTCTATTTGTATCGCTG- |
|  | *AmABCC-1-3189R* | CAGCGATACAAATAGAACTTGCC |
|  | *AmABCC-2-UTRF* | AACCAGTGACTCCTCCTCGTC |
|  | *AmABCC-2-UTRR* | AATCGAATTGCTCATG |
|  | *AmABCC-2-510F* | GTGAACTTGCTCCTCCTGCTC |
|  | *AmABCC-2-510R* | GAGCAGGAGGAGCAAGTTCAC |
|  | *AmABCC-2-1178F* | CTACATTGCCGTGGACGCTTA |
|  | *AmABCC-2-1178R* | TAAGCGTCCACGGCAATGTAG |
|  | *AmABCC-2-1864F* | CAGCCGATCTAGCACTAAGGAATG |
|  | *AmABCC-2-1864R* | CATTCCTTAGTGCTAGATCGGCTG |
|  | *AmABCC-2-2583F* | CATTGGTGTCGACACGGCAG |
|  | *AmABCC-2-2583R* | CTGCCGTGTCGACACCAATG |
|  | *AmABCC-2-3362F* | TTCCACACGGTTGCCGCACA |
|  | *AmABCC-2-3362R* | TGTGCGGCAACCGTGTGGAA |
|  | *AmABCC-2-4055F* | CTCGACACAGTAGTGAGTGATGA |
|  | *AmABCC-2-4055R* | TCATCACTCACTACTGTGTCGAG |
|  | *AmABCC-3-UTRF* | ACCGTCACCGAGTGAAGCTTC |
|  | *AmABCC-3-UTRR* | TTGATCTTCTAGCCTT |
|  | *AmABCC-3-506F* | GCTCACCCTGCGGCTATTCTG |
|  | *AmABCC-3-506R* | CAGAATAGCCGCAGGGTGAGC |
|  | *AmABCC-3-1328F* | AGATACACTACCTATGGCTCATGCC |
|  | *AmABCC-3-1328R* | GGCATGAGCCATAGGTAGTGTATCT |
|  | *AmABCC-3-2162F* | GATCCAGAACGGCACCATTGA |
|  | *AmABCC-3-2162R* | TCAATGGTGCCGTTCTGGATC |
|  | *AmABCC-3-3069F* | CAAGCAGATCCTCAACAGCATC |
|  | *AmABCC-3-3069R* | GATGCTGTTGAGGATCTGCTTG |
|  | *AmABCC-3-3739F* | CTGAGGCAGAATGGAGGATCA |
|  | *AmABCC-3-3739R* | TGATCCTCCATTCTGCCTCAG |
|  |  |  |
| qRT-PCR |  |  |
|  | *AmABCC-1-F* | GATCCGGACAGAATTCAAGCATTG |
|  | *AmABCC-1-R* | AGTACTCATTGACCAGATCGCG |
|  | *AmABCC-2-F* | TGATAACCATTGCTCACAGGGTC |
|  | *AmABCC-2-R* | TCATGTTGAGTTCCGCTTGCAA |
|  | *AmABCC-3-F* | CACTGCATCGGTGGATTCC |

**Supplementary Table S1** (continues)

| Type | Gene | Sense (5’-3’) |
| --- | --- | --- |
| qRT-PCR | *AmABCC-3-R* | TAGTCCTGCATCTATGACCAG |
|  | *AmGSTU2-F* | AAGCTTATCGTCGCGTGG |
|  | *AmGSTU2-R* | TCCAAAGTATCCCTGCCCCTT |
|  | *AmGSTF1-F* | AGTACGAGGTGGTGAACATCG |
|  | *AmGSTF1-R* | CCAGGTTGCTCTCCCTCAG |
|  | *AmABCB-1-F* | CTATGGTTGCTTCTGAGAGAGC |
|  | *AmABCB-1-R* | AGTGGTGATATTGGTGATTC |
|  | *AmABCB-2-F* | GCTGCGCAATGAAGCTGGATG |
|  | *AmABCB-2-R* | ACCGCGGCAGTGTCTTGTATG |
|  | *Am MATE1-F* | GGCCATCTTCGTCTACGG |
|  | *Am MATE1-R* | GTAGCTGAGGACGAGGTG |
|  | *Am MATE2-F* | CTGTGCTGCTCGCCTTCA |
|  | *Am MATE2-F* | CTGTGCTGCTCGCCTTCA |
|  | *Am MATE2-R* | AATACCGATGGGAACTCCAAC |
|  | *Am GSTF2-F* | ACGTGTGGCTGGAGGTTGAG |
|  | *Am GSTF2-F* | TTCCAGCACCTTCTTCAGCTTG |
| Reference |  |  |
|  | *AmG3DP-F* | ACTGATGACCACTGTTGATGC |
|  | *AmG3DP-R* | GACCATCCACAGTCTTCTGG |

**Supplementary Table S2** Amino acid sequences for generating blackgrass specific antisera for *Am*ABCC1, *Am*ABCC2 and *Am*GSTU2a

| Gene |  | Peptide sequence |
| --- | --- | --- |
|  |  |  |
| *Am*ABCC-1 | *Am*ABCC-1-1 | 1353-LEAVQEKEQGLDSL-1366 |
|  | *Am*ABCC-1-2 | 1412-LQKTIRTEFKHC-1423 |
| *Am*ABCC-2 | *Am*ABCC-2-1 | 1332-LKRISSTAALLDT-1344 |
|  | *Am*ABCC2-2 | 1439-LEDKQSAFAKL-1449 |
| AmGSTU2a | *Am*GSTU2a-1 | 141-GALRECSKGQGYF-153 |
|  | *Am*GSTU2a-2 | 175-SAVHSGIKIFDPIKTP-190 |

**Supplementary Table S3** A list of blackgrass populations used in this study. The herbicide resistance status of blackgrass populations have been confirmed by herbicide resistance assay and analysis of mutation in ALS or ACCase protein.

| Grass populations | Herbicide resistances |
| --- | --- |
| Blackgrass |  |
| Hor | TSR-ALS***** |
| Rothamsted (Roth) | Reference sensitive***** |
| Nott | TSR-ACCase***** |
| Warren | TSR-ALS, enhanced metabolic resistance***** |
| R30 | TSR-ALS, enhanced metabolic resistance***** |
| LongC | TSR-ALS, enhanced metabolic resistance***** |
| Velcourt (VelC) | TSR-ALS, enhanced metabolic resistance***** |
| Suffolk | NTSR field-collected***** |
| Peldon (NTSR1) | Reference NTSR***** |
| Pendimethalin-selected (NTSR2) | Enhanced metabolic resistance to ACCase herbicides^$^ |
| Fenoxaprop-selected (NTSR3) | Resistance to fenoxaprop without mutation or enhanced metabolic resistance^$^ |

* Marshall, R., Hanley, S.J., Hull, R. & Moss, S.R. (2012). The presence of two different target-site resistance mechanisms in individual plants of *Alopecurus myosuroides* Huds., identified using a quick molecular test for the characterisation of six ALS and seven ACCase SNPs. *Pest. Manag. Sci* **69**, 727-737.

^§^ Tétard-Jones, C., Sabbadin, F., Moss, S., Hull, R., Neve, P., and Edwards, R. (2018). Changes in the proteome of the problem weed blackgrass correlating with multiple-herbicide resistance. *Plant J.* **94**, 709–720.

**Supplementary Figures**

**
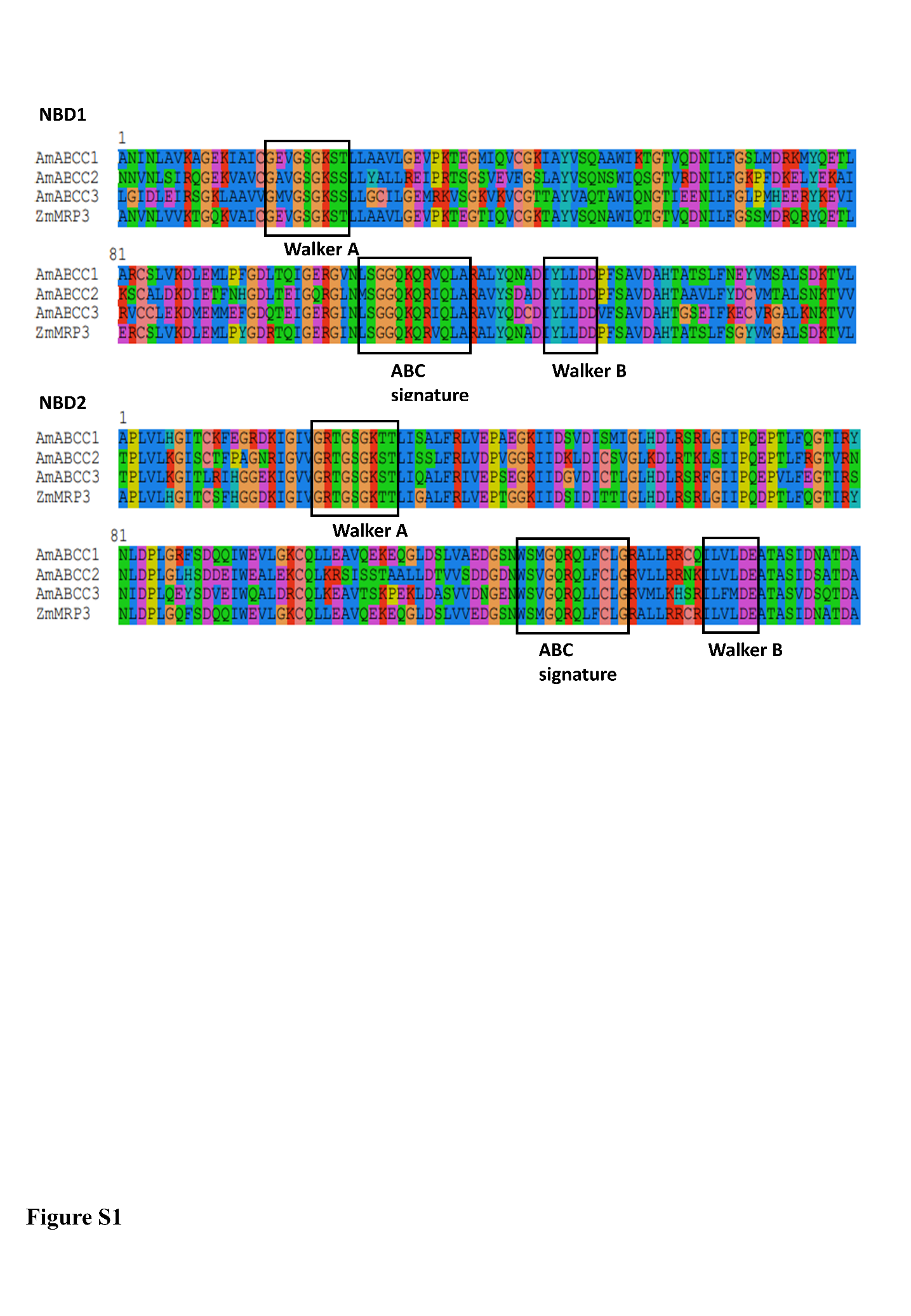
**

**Supplementary Figure S1** Amino acids sequences analysis of *Am*ABCC1, *Am*ABCC2, *Am*ABCC3 and *Zm*MPR3 proteins. The typical motif for ABC signature and ATP binding and hydrolysis (Walker A and B motifs) amino acid sequences were showed within the rectangles.

**
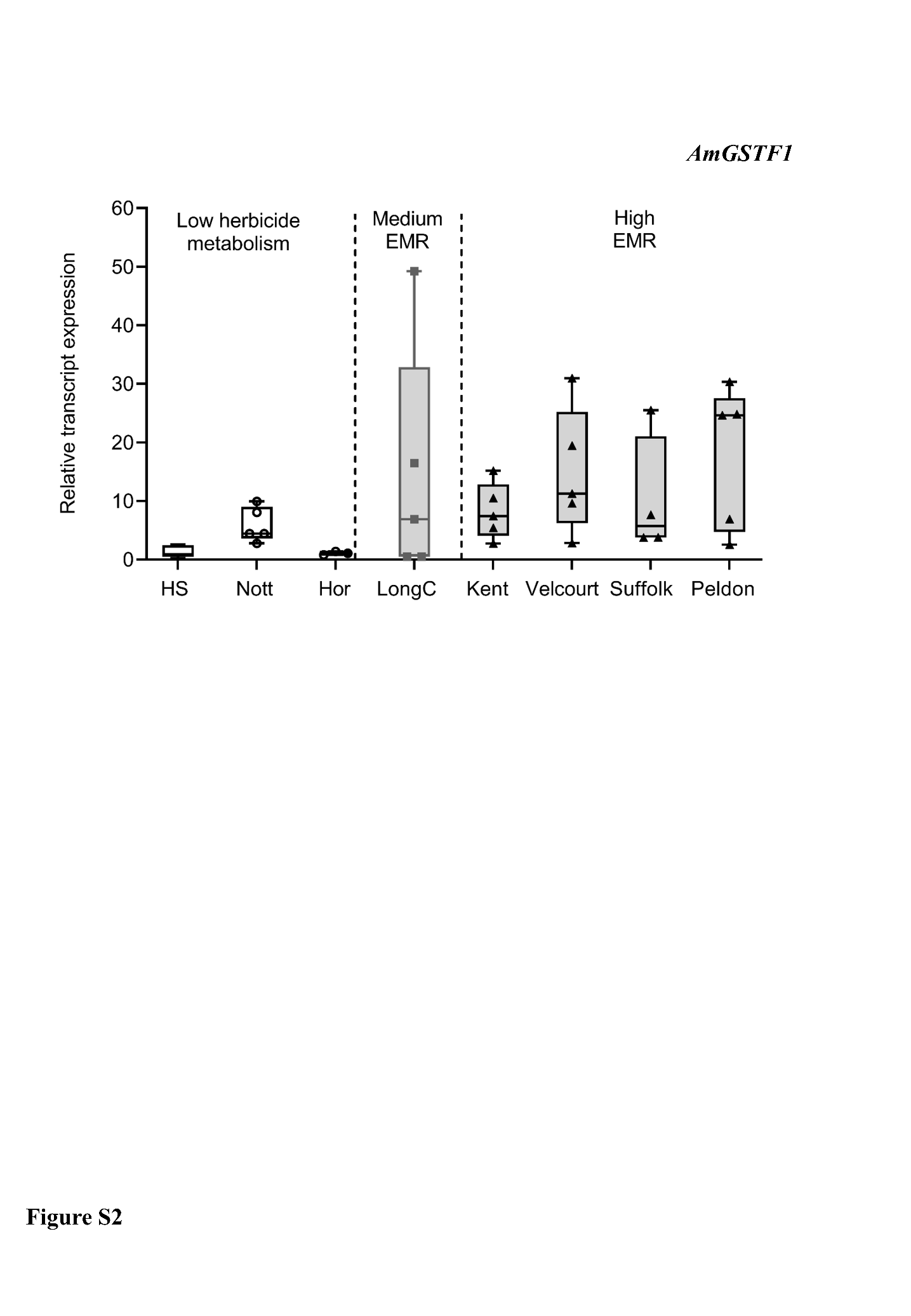
**

**Supplementary Figure S2** The relative transcript expression of *AmGSTF1* at the constitutive levels of field derived blackgrass populations collected across the UK. The herbicide sensitive (HS), target site mutation (Nott, Hor) and NTSR (LongC, Kent, Velcourt, Suffolk, Peldon) were used. Box plots represent relative transcript expression (n=3-5) in field-derived blackgrass populations of defined EMR phenotype.

**
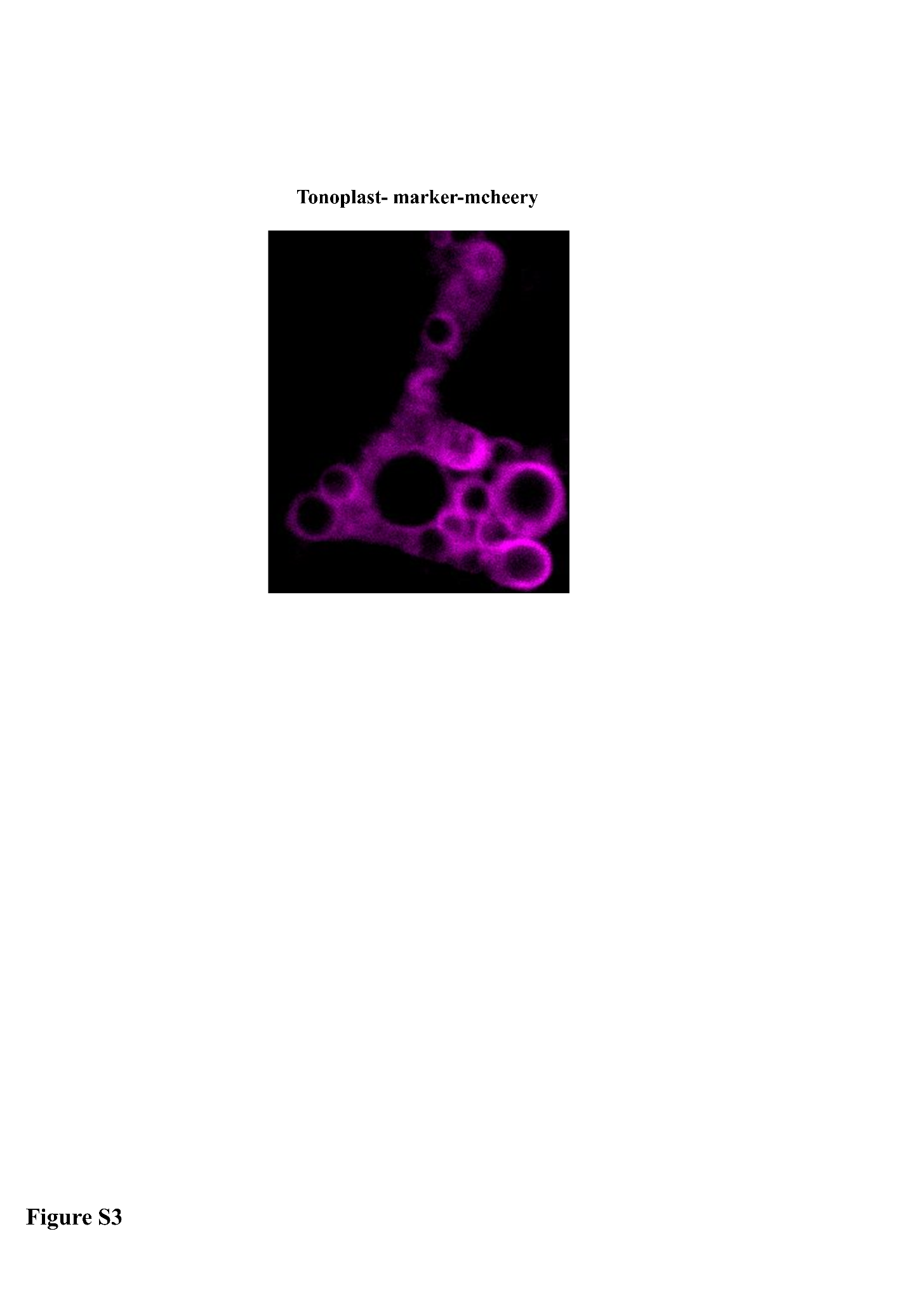
Supplementary Figure S3** The subcellular localization of the tonoplast marker (γ-TIP) fusion with m-cheery in *Nicotiana benthamiana* leaves

**
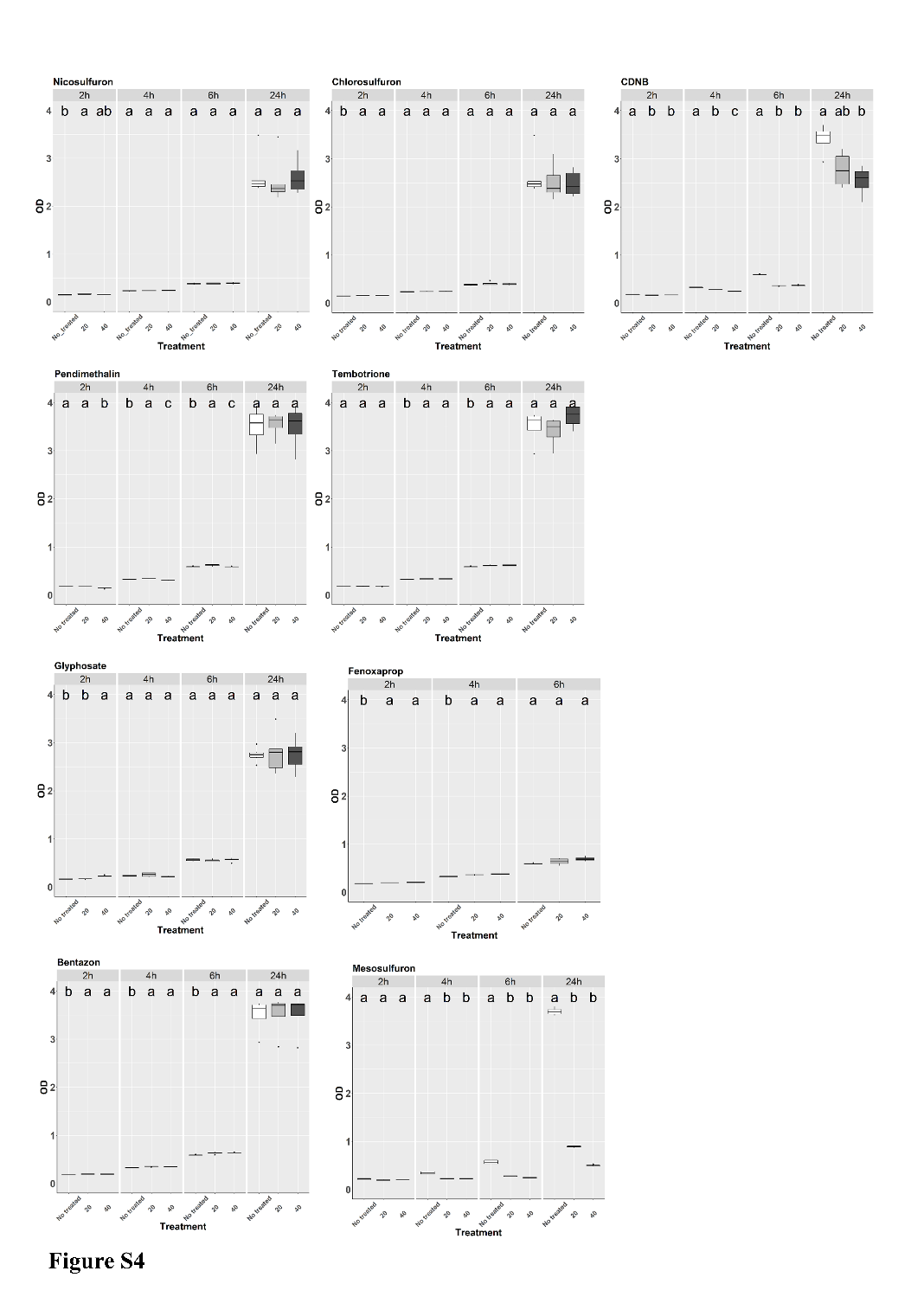
**

**Supplementary Figure S4** The effects of herbicides on yeast cell growth. *Am*ABCC was heterologous expressed in yeast cells before treated with nicosulfuron, chlorotoluron, CDNB, pendimethalin, tembotrione, glyphosate, fenoxaprop-ethyl, bentazone and mesosulfuron-methyl. The OD_600_ were determined at 2, 4, 6 and 24 h after treatment with either 20 µM or 40 µM herbicides.
